# Identification and Health Risks of an Emerging Means of Drug Use in Correctional Facilities

**DOI:** 10.1001/jamanetworkopen.2024.51951

**Published:** 2024-12-23

**Authors:** David Kuai, Liz Eneida Rivera Blanco, Alex Krotulski, Sara Walton, Max Denn, Byron Kelly, Emily Kiernan, Alaina Steck, Joseph Carpenter

**Affiliations:** 1 Department of Emergency Medicine, Emory University, Atlanta, Georgia; 2 The Center for Forensic Science Research & Education, Horsham, Pennsylvania; 3 NaphCare Inc, Birmingham, Alabama; 4 Grady Health System, Atlanta, Georgia

## Abstract

**Importance:**

Recreational use of drug-soaked paper strips (hereafter, strips) in correctional facilities poses a major public health risk owing to the diverse and potentially severe toxic effects of the substances they contain. Understanding the clinical manifestations and outcomes of exposure to these strips is important for developing effective management and prevention strategies.

**Objective:**

To characterize the clinical manifestations, management, and outcomes of intoxication from strips in a correctional facility population, and to identify the specific substances present in these strips.

**Design, Setting, and Participants:**

This is a case series with a retrospective medical record review and biospecimen analysis. Participants were incarcerated individuals from a county jail who presented to the emergency department of an urban hospital in Atlanta, Georgia, with suspected strip exposure between August 1, 2022, and November 1, 2023. Strip samples were tested using gas chromatography-mass spectrometry and liquid chromatography quadrupole time-of-flight mass spectrometry (LC-QTOF-MS), and serum samples were analyzed via LC-QTOF-MS.

**Exposures:**

All patients had suspected exposure to strips, thought to contain opioids, synthetic cannabinoid receptor agonists (SCRAs), and other novel synthetic drugs.

**Main Outcomes and Measures:**

The primary outcomes were clinical manifestations of strip exposure, including clinical signs and symptoms, vital sign derangements, laboratory and imaging findings, treatments administered, and patient outcomes.

**Results:**

There were 18 patients (all male; median [IQR] age, 27.5 [18.0-45.0] years) in total. Bradycardia (11 patients [61%]) and central nervous system depression (17 patients [94%]) were the most common clinical findings. Other symptoms included agitation (6 patients [33%]) and seizures (4 patients [22%]). Treatment primarily involved supportive care, with 2 patients requiring intubation. One patient died as a result of hypoxic ischemic encephalopathy. Analysis of strip samples revealed the presence of synthetic cannabinoids and benzimidazole opioids, but no household cleaners or pesticides. Serum testing confirmed the presence of SCRAs and their metabolites.

**Conclusions and Relevance:**

In this case series study of strip intoxication among incarcerated individuals, severe and variable clinical manifestations were observed, predominantly associated with their SCRA content. Bradycardia in the context of major central nervous system depression was an indicator of severe SCRA toxic effects. Further research is needed to better understand strip use and to develop effective prevention and treatment strategies.

## Key Points

**Question:**

What are the clinical manifestations, management, and outcomes associated with exposure to drug-soaked paper strips among incarcerated individuals, and which substances are present in these strips?

**Findings:**

In this case series of 18 male patients with strip intoxication, clinical manifestations included central nervous system depression (94%) and bradycardia (61%). Strips primarily contained synthetic cannabinoid receptor agonists, nitazene opioids, and other novel psychoactive substances.

**Meaning:**

These findings suggest that drug-soaked paper strips can cause severe health outcomes, and improved detection, management strategies, and further research are needed to understand this emerging public health threat.

## Teaser

This case series discusses the clinical manifestations, management, and outcomes of intoxication from drug-soaked paper strips in a correctional facility population and identifies the specific substances present in these paper strips.

## Introduction

The emergence of novel methods and substances used recreationally within correctional facilities is an area of growing public health concern. According to a report released by the Bureau of Justice Statistics, a federal agency in the US Department of Justice, drug and/or alcohol intoxication deaths in state prisons increased by more than 600% from 2001 to 2018.^1^ The use of drug-soaked paper strips (hereafter, strips) has gained attention as an increasing trend, often requiring transfer to the hospital for medical care. Strips are pieces of paper that are sprayed with or soaked in liquid formulations of various drugs, suspected to be opioids, synthetic cannabinoid receptor agonists (SCRAs),^2,3^ and other novel synthetic drugs. Use of strips is a public health concern because of the heterogeneous nature of the drugs and the unique risk of so-called hot spots, where concentrations of the drug are higher in certain areas of the paper owing to uneven application or pooling during the drying process, thus increasing the risk of overdose.^4^ It is not uncommon for a group of inmates to ingest or smoke strips from the same sheet of paper, with only a subset experiencing adverse health outcomes.

A prototypical method of smuggling strips into correctional facilities involves individuals outside posing as attorneys and sending drug-soaked 8.5- by 11.0-inch pieces of paper interspersed with normal paper to certain inmates. Mail from attorneys to inmates is confidential and generally cannot be opened or inspected by facility staff. After the drug-soaked papers are in the inmates’ possession, the pieces of paper are divided into small strips and smoked or consumed orally.

Use of strips has been reported in many states across the US,^4,5,6^ as well as in countries in Europe. Samples from Finland, Germany, Hungary, Lithuania, Poland, Sweden, the United Kingdom,^7^ and Brazil^8^ have revealed the presence of SCRAs.^6,9^ According to inmates, strips contain household products such as household cleaning agents, rodenticides, and other pyrethroid-containing pesticides,^10,11^ which have been used for recreational purposes in the past and have variable clinical manifestations.^12^ The terms *roach spray* and *wasp spray* are commonly associated with drug use in correctional facility settings in the US, but findings of the true substances associated with these drugs are rare.

Reports on the content of strips vary widely according to accounts from inmates, the media, and the literature reported abroad.^7,8,9,13^ Some of the existing global literature characterizes strips as containing SCRAs or opioids. The manifestations of these substances are well-described.^3^ However, to our knowledge, neither the content of strips used in correctional facilities in the US nor the clinical outcomes of this exposure have been documented. Therefore, the purpose of this study is to describe the presentation, clinical course, management, and outcomes of patients exposed to strips at a single hospital. Furthermore, we aim to characterize the substances found on strips and confirm the exposures using comprehensive serum testing.

## Methods

### Study Design

This is a retrospective case series with an electronic medical record (EMR) review and secondary biospecimen analysis. The study population includes patients from a local county jail who presented to the Grady Memorial Hospital (Atlanta, Georgia) emergency department (ED) with suspected exposure to strips. All adult patients (≥18 years old) with this suspected exposure from August 1, 2022, to November 1, 2023, whose cases were reported to the Georgia Poison Center were included. Cases were excluded if there was both a low clinical suspicion for intoxication due to strips and laboratory exclusion of psychoactive substances. The study was conducted in compliance with all applicable ethical guidelines. The Emory University institutional review board reviewed and approved the study, including specific considerations regarding research involving incarcerated individuals, and granted a waiver of consent, in accordance with 45 CFR §46. This report follows the Strengthening the Reporting of Observational Studies in Epidemiology (STROBE) reporting guidelines.

### EMR Review

We extracted data from EMRs, including demographic information (age, legal sex, and medical history), prehospital and in-hospital symptoms, physical examination findings, vital signs, abnormal laboratory values, imaging findings, treatment modalities, patient disposition, and patient outcomes. Vital signs obtained included the peak and nadir vital signs (temperature, pulse, respiratory rate, blood pressure, and oxygen saturation) within 6 hours of ED arrival. Hypothermia was defined as a body temperature of less than 95 °F (35 °C), bradycardia as a pulse rate less than 60 beats per minute, tachycardia as a pulse rate greater than 100 beats per minute, hypoxia as an oxygen saturation of less than 90% on room air, and hypotension as a mean arterial pressure less than 65 mm Hg or a systolic blood pressure less than 100 mm Hg.

### Data Abstraction

Two investigators (D.K. and L.E.R.B.) independently reviewed each EMR and produced complete datasets, which were then cross-checked for consistency. Discrepancies were jointly reviewed until a consensus was reached.

### Laboratory Evaluation

Two batches of strip samples were provided by the local county jail (Atlanta, Georgia) where this outbreak occurred. Strip samples were obtained in the same period as biologic samples; however, the exact strips used by patients included in the study were not available for testing. Waste biological samples from patients with suspected strip intoxication were obtained from the hospital laboratory. Strip and waste biological samples were sent to a reference laboratory for comprehensive drug testing (Center for Forensic Science Research & Education, Horsham, Pennsylvania).

Drug materials (ie, paper strips) were diluted in solvent and prepared by acidic and basic liquid-liquid extraction. Samples were analyzed by gas chromatography mass spectrometry and liquid chromatography quadrupole time-of-flight mass spectrometry (LC-QTOF-MS). Data files were processed against an in-house library database containing more than 1100 targets, including recreational drugs, therapeutic substances, novel psychoactive substances, and metabolites. Toxicology specimens (eg, blood and serum or plasma) were prepared by basic and acidic liquid-liquid extractions to isolate drugs from matrix. Samples were analyzed by LC-QTOF-MS, and data files were processed in an identical manner to drug material samples.

### Statistical Analysis

Demographic data, clinical signs and symptoms, treatment modalities, and patient outcomes are presented descriptively. Categorical variables are presented as frequencies and percentages, and continuous variables are presented as medians with IQRs. We assessed certain parameters such as blood pressure as both a categorical and continuous variable. No formal hypothesis testing was conducted because of the retrospective method and small sample size. Data were managed with Excel 365 (Microsoft).

## Results

Nineteen patients met the inclusion criteria. One patient was excluded retrospectively because of low clinical suspicion of strip intoxication following EMR review by the study team and laboratory exclusion of any xenobiotics present in serum via comprehensive screening. Exclusion was agreed upon by all reviewers. Eighteen patients (all male; median [IQR] age, 27.5 [18.0-45.0]) were included in the final analysis (Table 1).

**Table 1.** Characteristics of Patients With Clinical Intoxication From Drug-Soaked Paper Strips

Characteristic	Patients, No. (%)
Male sex	18 (100)
Age, median (IQR), y	27.5 (18.0-45.0)
Vital signs	
Nadir heart rate, median (IQR), beats/min	57 (50-61)
Hypotension present	7 (39)
Hypoxia present	6 (33)
Hypothermia present	4 (22)
Signs and symptoms	
Central nervous system depression	17 (94)
Agitation and/or combativeness	6 (33)
Seizures	4 (22)
Nausea and/or vomiting	3 (17)
Cardiac arrest	1 (6)
Glasgow Coma Scale score, median (IQR)^a^	
At jail	8 (7-13)
At emergency department presentation	14 (13-15)

^a^ The Glasgow Coma Scale ranges from 3 (unresponsive) to 15 (responsive; ie, eyes open spontaneously, oriented, and following commands).

### Signs and Symptoms

The most common vital sign derangement was bradycardia, seen in 11 of 18 patients (61%). The median (IQR) nadir heart rate among all patients was 57 (50-61) beats per minute. Three patients experienced tachycardia, and the median (IQR) peak heart rate among all patients was 85 (71-96) beats per minute. Several patients also experienced hypotension (7 patients [39%]), hypoxia either before ED presentation or in the ED (6 patients [33%]), and/or hypothermia (4 patients [22%]; core temperature range among hypothermic patients, 89-92 °F). One hypothermic patient arrived in pulseless electrical activity arrest after at least 35 minutes of prehospital cardiopulmonary resuscitation.

All patients except 1 experienced central nervous system (CNS) depression (17 patients [94%]). The patient who did not experience CNS depression developed agitation. Patients had CNS depression either at the jail (17 patients [94%]) or upon arrival at the ED (11 patients [61%]). Patients had a median (IQR) Glasgow Coma Scale (GCS) score of 8 (7-13) at the jail and 14 (13-15) upon presentation to the ED (the GCS ranges from 3 [unresponsive] to 15 [eyes open spontaneously, oriented, and following commands]). Four patients (22%) had 1 or more seizures, 1 of whom developed status epilepticus.

The patient who arrived in pulseless electrical activity arrest died, and 1 patient experienced multiple seizures, hypoxia, and required intubation. Three patients with seizures had elevated lactic acid levels (17%). There were 2 patients with elevated high-sensitivity troponin. One patient with psychomotor agitation experienced elevated creatine phosphokinase levels. There was no specific pattern of laboratory findings observed. On chest imaging, 4 patients had findings suspicious for aspiration, 1 for pulmonary edema, and 1 for nonspecific atelectasis.

### Treatments and Case Outcomes

The most frequent prehospital treatment was naloxone (14 patients [78%]) (Table 2). Two of the 14 patients received naloxone via an unknown route. All other patients who received prehospital naloxone received it via the intranasal route, at a dose ranging from 4 to 8 mg. Of the patients who received naloxone, 3 of 14 had a response, although the exact response was not documented. One patient received an additional 8 mg intravenous (IV) dose prehospital without a response, and 1 patient received a 0.2 mg IV dose in-hospital with no response. Atropine was given to 2 patients prehospital and 1 in the hospital. There was a documented increase in heart rate and blood pressure in both cases, and improvement in GCS score from 3 to 12 in 1 patient who received atropine prehospital. The most common treatments in the hospital included IV fluids (16 patients [89%]) and benzodiazepines (5 patients [28%]). All 4 hypothermic patients were treated using a forced-air blanket; 2 received warm IV fluids. Two patients (11%) were intubated: 1 with acute hypoxic respiratory failure and status epilepticus, and 1 who was found in cardiac arrest.

**Table 2.** Prehospital and In-Hospital Treatment, Disposition, and Medical Outcomes of Study Patients

Variable	Patients, No. (%)
Prehospital treatment	
Naloxone	14 (78)
Atropine	2 (11)
Intravenous fluids	2 (11)
Sedative medications	2 (11)
Hospital treatment	
Intravenous fluids	16 (89)
Sedative medications	6 (33)
Intubation	2 (11)
Disposition	
Discharge	7 (39)
Admission to floor	8 (44)
Admission to critical care unit	3 (17)
Medical outcome	
Survival	17 (94)
Death	1 (6)

Seven patients (39%) were discharged from the ED. Eight patients (44%) were admitted to a general medical floor, and 3 (17%) were admitted to the intensive care unit. There was 1 death (6%), in a patient who was found down in the jail cell pulseless and apneic. Return of spontaneous circulation was achieved after an extended resuscitation; however, he experienced hypoxic-ischemic encephalopathy and was transitioned to comfort care.

### Forensic Laboratory Analyses

Fourteen total strip samples in 2 batches were analyzed and found to contain numerous synthetic drugs (Table 3 and eFigure in Supplement 1). Photographs of the physical samples are displayed in the Figure. Major components of the strip samples were indazole-based synthetic cannabinoids, including MDMB-4en-PINACA and/or AB-CHMINACA. Minor and trace components of the samples included indazole precursor SCRA compounds (eg, MDMB-INACA and ADB-INACA), other indazole-based and non–indazole-based SCRAs (eg, ADB-BINACA, ADB-4en-PINACA, 4F-MDMB-BINACA, MDMB-5Me-INACA, and UR-144), 2 benzimidazole opioids, also known as nitazene analogs (eg, metonitazene and 5-methyl etodesnitazene), hallucinogen-type arylcyclohexylamines (eg, 2F-2oxo-PCE and 2F-deschloronorketamine), as well as more common substances (eg, fentanyl, methamphetamine, xylazine, and caffeine). Despite initial inmate reports of the strips containing various household cleaners and pesticides, these substances were not detected on any strip samples analyzed at the Center for Forensic Science Research & Education.

**Table 3.** Results of Testing of Physical Strip Samples

Sample and paper No.^a^	Components^b^	
	Major	Minor
Sample 1		
Paper 1	MDMB-4en-PINACA (1p)	ADB-BINACA (minor), ADB-4en-PINACA (minor), MDMB-INACA (precursor), ADB-INACA (precursor)
Paper 2	MDMB-4en-PINACA (1p)	ADB-BINACA (minor), ADB-4en-PINACA (minor), MDMB-INACA (precursor), ADB-INACA (precursor)
Paper 3	MDMB-4en-PINACA (1p)	None
Sample 2		
Paper 1	MDMB-4en-PINACA (1p)	ADB-BINACA (minor), ADB-4en-PINACA (minor), 4F-MDMB-BINACA (minor), MDMB-INACA (precursor), ADB-INACA (precursor)
Paper 2	MDMB-4en-PINACA (1p)	ADB-BINACA (minor), ADB-4en-PINACA (minor), 4F-MDMB-BINACA (minor), MDMB-INACA (precursor), ADB-INACA (precursor)
Paper 3	MDMB-4en-PINACA (1p)	None
Sample 3		
Paper 1	MDMB-4en-PINACA (1p)	ADB-BINACA (minor), ADB-4en-PINACA (minor), MDMB-INACA (precursor), ADB-INACA (precursor)
Paper 2	MDMB-4en-PINACA (1p)	MDMB-INACA (precursor)
Paper 3	MDMB-4en-PINACA (1p)	MDMB-INACA (precursor)
Sample 4		
Paper 1	MDMB-4en-PINACA (1p), AB-CHMINACA (0.8p)	2F-2oxo-PCE (0.2p), ADB-4en-PINACA (0.1p), metonitazene (trace), MDMB-INACA (trace), fentanyl (trace), MDMB-5Me-INACA (trace), UR-144 (trace)
Paper 2	MDMB-4en-PINACA (1p)	ADB-4en-PINACA (0.3p), MDMB-INACA (0.2p), MDMB-5Me-INACA (0.1p), ADB-INACA (trace), 2F-2oxo-PCE (trace), methamphetamine (trace), 2F-deschloronorketamine (trace)
Paper 3	MDMB-4en-PINACA (1p)	AB-CHMINACA (0.3p), 2F-2oxo-PCE (0.1p), ADB-4en-PINACA (0.1p), metonitazene (trace), MDMB-INACA (trace), methamphetamine (trace), fentanyl (trace), 2F-deschloronorketamine (trace), xylazine (trace)
Sample 5	MDMB-4en-PINACA (1p), AB-CHMINACA (0.6p)	ADB-4en-PINACA (0.1p), 2F-2oxo-PCE (0.1p), metonitazene (trace), MDMB-INACA (trace), UR-144 (trace), MDMB-CHMINACA (trace), 5-methyl etodesnitazene (trace)
Sample 6	MDMB-4en-PINACA (1p), AB-CHMINACA (0.6p)	ADB-4en-PINACA (0.1p), 2F-2oxo-PCE (trace), metonitazene (trace), MDMB-INACA (trace), UR-144 (trace), 5-methyl etodesnitazene (trace), caffeine (trace)
Sample 7, paper 1	MDMB-4en-PINACA (1p), AB-CHMINACA (0.5p)	2F-2oxo-PCE (0.1p), ADB-4en-PINACA (trace), metonitazene (trace), MDMB-INACA (trace), UR-144 (trace), caffeine (trace)

^a^ Samples are defined as packages containing multiple sheets of paper, some of which are soaked with drug product (see Figure). Papers are defined as sampled papers within each sample. Samples 1 to 3 were sent from a batch of strips obtained in 2023, whereas samples 4 to 7 were sent from a batch obtained in 2024.

^b^ Substances detected were separated into major and minor components designations based on instrumental peak area. Respective ratios of the substances are designated in parts (p); for example, A (1p) and B (0.5p) signifies component B is present at half the amount of component A. Components less than 10% of the major component (ie, <0.1p) are designated as minor or trace.

**Figure.**
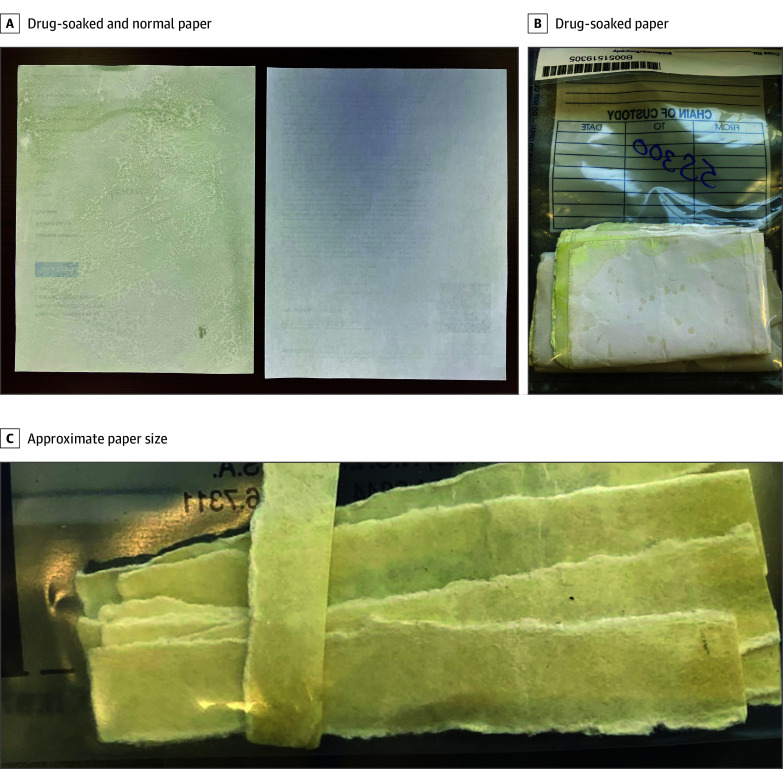
Photographs of Drug-Soaked Paper Strip Samples Packages of intercepted mail addressed to inmates forged to appear from attorney’s offices were seized by a county jail and sent for testing. The papers appear to be miscellaneous articles gathered online and made to superficially appear to be legal documents. Panel A shows the difference in appearance from a drug-soaked piece of paper (left) vs a normal, non–drug-soaked piece of paper (right). In some cases, drug-soaked pieces of paper appeared very similar visually but had a textural difference from the regular pieces of paper. Panel B shows a close-up of the drug-soaked pieces of paper. Panel C shows a close-up of the approximate size of strip of paper when used recreationally.

Waste serum samples were available for 13 of 19 patients. The patient with low suspicion of strip exposure had serum testing excluding the presence of psychoactive substances. Although the physical strip samples tested were not the exact strip samples used by the patients, recreational drugs found in serum testing of all other tested patients included SCRAs found on strip sample testing and SCRA metabolites (Table 4). Other substances detected by testing of the strip samples were not detected in patient serum samples, likely due to low levels falling below toxicologically relevant limits of detections. The in-house LC-QTOF-MS library database used for processing samples is included in the eTable in Supplement 1.

**Table 4.** Comprehensive Toxicology Results Obtained From Serum Specimens Tested

Serum sample No.	Detected substances and metabolites
1	MDMB-4en-PINACA 3,3-dimethylbutanoic acid
2	MDMB-4en-PINACA 3,3-dimethylbutanoic acid
3	AB-CHMINACA 3-methylbutanoic acid
4	MDMB-4en-PINACA, AB-CHMINACA, MDMB-4en-PINACA 3,3-Dimethylbutanoic Acid
5	Methamphetamine, MDMB-4en-PINACA 3,3-Dimethylbutanoic Acid, AB-CHMINACA 3-Methylbutanoic Acid
6	MDMB-4en-PINACA 3,3-dimethylbutanoic acid
7	MDMB-4en-PINACA 3,3-dimethylbutanoic acid, AB-CHMINACA 3-methylbutanoic acid
8	MDMB-4en-PINACA 3,3-dimethylbutanoic acid, AB-CHMINACA 3-methylbutanoic acid
9	MDMB-4en-PINACA, MDMB-4en-PINACA 3,3-dimethylbutanoic acid
10	AB-CHMINACA 3-methylbutanoic acid, MDMB-4en-PINACA 3,3-dimethylbutanoic acid
11	MDMB-4en-PINACA, MDMB-4en-PINACA 3,3-Dimethylbutanoic Acid, AB-CHMINACA 3-methylbutanoic acid, quinine
12	MDMB-4en-PINACA 3,3-dimethylbutanoic acid
13	AB-CHMINACA, AB-CHMINACA 3-methylbutanoic acid
14	AB-CHMINACA, AB-CHMINACA 3-methylbutanoic acid
15	AB-CHMINACA, AB-CHMINACA 3-methylbutanoic acid

## Discussion

The findings of this case series provide insights into the variable clinical presentations associated with the misuse of strips among incarcerated individuals. The identification of various SCRAs, nitazene opioids, and other substances within strips underscores the polydrug nature of this clinical scenario, contributing to the diverse and occasionally severe clinical manifestations observed. Serum testing did not detect other xenobiotics (ie, opioids or hallucinogens) seen in the tested strips. We hypothesize that this was due to their trace presence on the strips and, therefore, very low concentrations in serum, likely below the detection limits of the testing assays, which are set according to toxicological relevance and analytical capabilities (most drug detection limits are <1 ng/mL). All of the tested and included patients had SCRAs and SCRA metabolites detected in serum. Agitation, seizures, and CNS depression, which were seen in patients in this study, are common manifestations of SCRAs.^14^ This finding suggests that the primary components of strips causing intoxication were likely the SCRAs, although biologic testing was limited to serum and the pharmacokinetics of some of the other detected substances are poorly understood.

Bradycardia was a common finding in patients in this study, which stands in contrast to a typical patient with SCRA intoxication who has tachycardia and agitation.^15,16^ It has been hypothesized that bradycardia is a symptom of severe cannabinoid toxic effects and that δ-9-tetrahydrocannabinol has a biphasic, dose-dependent effect on the cardiovascular system, causing predominantly sympathetic activity at low-to-moderate doses and parasympathetic activity at higher doses.^17^ Strong activation of presynaptic cannabinoid type 1 (CB1) receptors in the cardiac tissues may lead to inhibition of norepinephrine release from associated synapses and subsequent bradycardia.^18^ A previous study of patients who used nonsynthetic cannabinoid products showed that patients experiencing bradycardia also had substantial sedation.^16^ Bradycardia also appears to be correlated with CNS sedation in patients with intoxication from SCRAs. This may be due to the full agonist activity of SCRAs at the cannabinoid receptors and has been demonstrated in a small case series involving SCRA intoxication.^19^ In our study, all patients experienced substantial sedation at the jail or upon arrival to the ED, and the majority of patients experienced bradycardia. All patients with a GCS of 8 or lower upon ED arrival were documented to have bradycardia (except the patient who arrived in cardiac arrest). The major components of the drug samples tested were potent full agonists at CB1, and most synthetic cannabinoids except UR-144 are also strong CB1 full agonists. Bradycardia could also be attributed to other drugs that may not have been detected due to rapid elimination or otherwise poorly understood kinetics.

Hypothermia was seen in several patients, which is uncommonly reported in cases of SCRA intoxication. Clinical reports have reported it as a rare manifestation of nonsynthetic cannabinoids,^20^ and it has been described in various animal models after SCRA exposure.^21^ The exact mechanism of SCRA-induced hypothermia is poorly understood in humans. CB1 receptors and endocannabinoids are present in the hypothalamus and are thought to contribute to overall thermoregulation through complex mechanisms.^22^ Hypothermia may also be related to opioid agonists and/or hallucinogens detected in many of the samples.

Because most clinical manifestations of strips can likely be attributed to their SCRA content, overall management is tailored toward treatment for severe SCRA toxic effects and is supportive in nature. The mainstay of management for psychomotor agitation, as well as for seizures from SCRA use, is benzodiazepines, which help prevent rhabdomyolysis and other complications. Patients who develop rhabdomyolysis should receive IV fluids. Patients who have agitation or seizures refractory to typical doses of benzodiazepines may require deeper sedation and endotracheal intubation.^23^ Atropine may be a reasonable choice for patients with severe SCRA toxic effects who present with hypotension and bradycardia. All patients had an increase in blood pressure and heart rate in response to atropine, and 1 patient also had improvement in his mental status.

Three patients were reported to respond to naloxone despite an absence of opioids on serum testing. Response to naloxone may have been due to reversal of manifestations of other trace opioids observed on strip testing, and it is reasonable to administer naloxone to patients with intoxication from strips who have respiratory depression. Because of the heterogeneous content of strips, the overall treatment of intoxicated patients should be based on the clinical signs and symptoms observed. As we noted here, the clinical presentation can be quite variable, and treatment will vary between patients.

Prevention of strips use in correctional facilities requires a multifaceted approach that includes intervention by both correctional facility staff and health care practitioners. Traditional methods of detecting substances smuggled into correctional facilities involves cell, inmate, or visitor searches by staff and use of trained drug detection dogs. Detection of smuggled strips may require more complex methods, including in-field analytical techniques such as ion mobility spectrometry or analysis of samples by external forensic laboratories.^24^ Addressing underlying reasons for drug use from a health care standpoint can also be beneficial. This includes providing access to mental health services and substance use disorder treatment.^25^

Future studies should focus on several key areas to better understand and mitigate the risks associated with strips use in correctional facilities. First, larger multicenter studies are needed to validate the findings of this initial case series, which may elucidate regional differences in clinical manifestations and outcomes of strip use. Second, research should aim to quantify drug concentrations of specific samples used by patients and correlate these with clinical outcomes. This would help in understanding dose-response relationships and could inform treatment protocols and further studies on pharmacokinetics and pharmacodynamics of the substances found on strips to better predict their clinical outcomes. Finally, long-term health outcomes of strips use are currently unknown, and longitudinal studies may be of benefit to evaluate long-term risks such as physical dependence and withdrawal. By addressing these areas, future research can contribute to a comprehensive strategy for managing the public health threat posed by strip use in correctional facilities.

### Limitations

This study has several limitations. The retrospective design and reliance on available EMRs may introduce biases. The small sample size and single-center design restrict the generalizability of the findings. In addition, the exact doses of the substances used could not be determined, and quantity of drugs on the physical strip samples could not be determined at the time of analysis, which could provide more precise correlations between substance concentrations and clinical outcomes. Only qualitative serum testing was performed; metabolites more frequently detected in urine samples may have been missed. Pharmacokinetics and pharmacodynamics of some drugs are poorly understood,^26^ and minor or trace substances detected on physical samples that may have caused clinical manifestations could have been metabolized by the time blood was drawn. Furthermore, there was no clear pattern between compounds detected in serum and the severity of symptoms experienced by patients. To this end, quantitative rather than qualitative testing may be more revealing in future studies. Future prospective studies with broad geographic coverage including larger sample sizes and more detailed substance quantification are needed to validate and expand upon these findings.

## Conclusions

In this case series study of strip intoxication among incarcerated individuals, we identified predominantly SRCAs on physical strip samples from a correctional facility setting, and serum specimens primarily contained SCRAs and their metabolites. Use of these chemical agents caused severe clinical manifestations, including 1 death. Improved detection, management strategies, and further research are needed to understand this emerging public health threat.

## Notes

Supplement 1. **eFigure.** Chemical structures of drugs detected on physical samples of paper strips **eTable.** CFSRE LC-QTOF-MS scope of testing

Supplement 2.
Data Sharing Statement
