# Supplementary material for: Identification and Health Risks of an Emerging Means of Drug Use in Correctional Facilities
Source: JAMA Netw Open. 2024 Dec 23;7(12):e2451951. doi: 10.1001/jamanetworkopen.2024.51951 (PMC11667344; doi:10.1001/jamanetworkopen.2024.51951)
Supplement: Supplement 1. — eFigure. Chemical structures of drugs detected on physical samples of paper strips eTable. CFSRE LC-QTOF-MS scope of testing [file jamanetwopen-e2451951-s001.pdf]

Supplemental Online Content

Kuai D, Rivera Blanco LE, Krotulski A, et al. Identification and health risks of an emerging means of drug use in correctional facilities. *JAMA Netw Open*. 2024;7(12):e2451951. doi:10.1001/jamanetworkopen.2024.51951

eFigure. Chemical structures of drugs detected on physical samples of paper strips

eTable. CFSRE LC-QTOF-MS scope of testing

This supplemental material has been provided by the authors to give readers additional information about their work.

© 2024 Kuai D et al. *JAMA Network Open.*

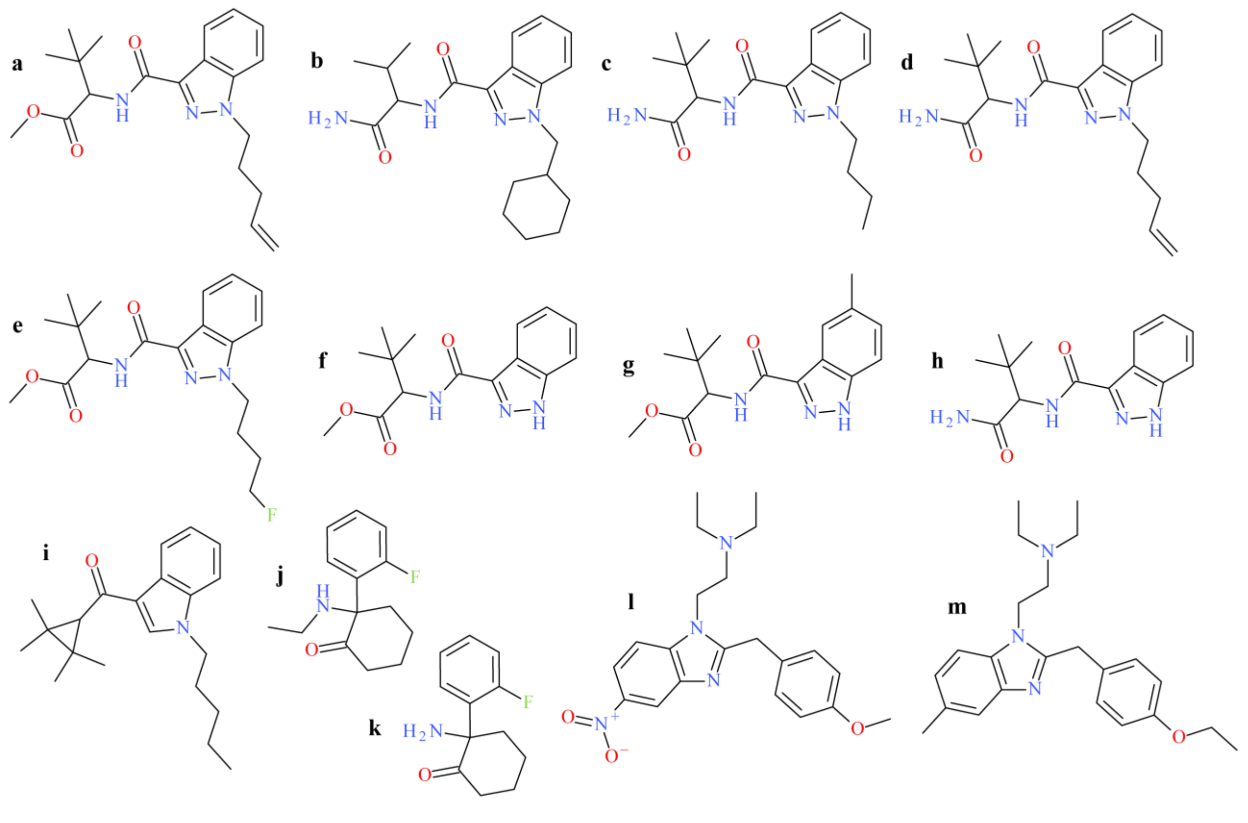

eFigure. Chemical structures of drugs detected on physical samples of paper strips. Figures 1a – 1e: MDMB-4en-PINACA, AB-CHMINACA, ADB-BINACA (also known as ADB-BUTINACA), ADB-4en-PINACA, 4F-MDMB-BINACA; indazole-based synthetic cannabinoids. Figures 1f – 1h: MDMB-INACA, MDMB-5Me-INACA, ADB-INACA; precursors for synthesis of indazole-based synthetic cannabinoids. Figure 1i: UR-144, a tetramethylcyclopropyl-based synthetic cannabinoid. Figure 1j – 1k: 2F-2oxo-PCE, 2-fluoro deschloronorketamine; arylcyclohexylamines (similar to phencyclidine, ketamine, etc.). Figure 1l– 1m: metonitazine, 5-methyl etodesnitazene; benzimidazole opioids (i.e., nitazene analogues). Not pictured but also detected included methamphetamine, caffeine, xylazine, and fentanyl. For a full list of detected substances, refer to **Table 3.**

© 2024 Kuai D et al. *JAMA Network Open.*

eTable. CFSRE LC-QTOF-MS scope of testing

Drug Name (Alphabetical)	Chemical Formula	Exact Mass (Da)
(6aR,9R)-delta-10-THC	C21H30O2	315.2319
1-(2,6-Xylyl)-2-Thiourea	C9H12N2S	181.0794
1-(4-methylbenzyl) piperazine	C12H18N2	191.1543
1,2,3,4-THBC	C11H12N2	173.1073
10-Hydroxycarbazepine	C15H14N2O2	255.1128
1B-LSD	C24H31N3O2	394.2489
1cP-LSD	C24H29N3O2	392.2333
1cP-MiPLA	C24H29N3O2	392.2333
1-Hydroxymidazolam	C18H13ClFN3O	342.0804
1P-LSD	C23H29N3O2	380.2333
2,3,4-Trimethoxyamphetamine	C12H19NO3	226.1438
2,3,5-Trimethoxyamphetamine	C12H19NO3	226.1438
2,3,6-Trimethoxyamphetamine	C12H19NO3	226.1438
2,4,5-Trimethoxyamphetamine	C12H19NO3	226.1438
2,4,6-Trimethoxyamphetamine	C12H19NO3	226.1438
2,5-Dimethoxy-4-Chloroamphetamine (DOC)	C11H16ClNO2	230.0942
2',5'-Dimethoxyfentanyl	C24H32N2O3	397.2486
2,6-Xylidine	C8H11N	122.0964
25B-NBF	C17H19BrFNO2	368.0656
25B-NBOH	C17H20BrNO3	366.0699
25B-NBOMe	C18H22BrNO3	380.0856
25C-NBF	C17H19ClFNO2	324.1161
25C-NBOH	C17H20ClNO3	322.1205
25C-NBOMe	C18H22ClNO3	336.1361
25D-NBOMe	C19H25NO3	316.1907
25E-NBOH	C19H25NO3	316.1907
25E-NBOMe	C20H27NO3	330.2064
25H-NBOMe	C18H23NO3	302.1750
25I-NBF	C17H19FINO2	416.0517
25I-NBOH	C17H20INO3	414.0561
25I-NBOMe	C18H22INO3	428.0717
25N-NBOMe	C18H22N2O5	347.1602
25T2-NBOMe	C19H25NO3S	348.1628
25T4-NBOMe	C21H29NO3S	376.1941
25T7-NBOMe	C21H29NO3S	376.1941
2Br-Deschloroketamine	C13H16BrNO	282.0488
2Br-DMPEA	C10H14BrNO2	260.0281
2C-B	C10H14BrNO2	260.0281
2C-B-FLY	C12H14BrNO2	284.0281
2C-C	C10H14ClNO2	216.0786
2C-D	C11H17NO2	196.1332
2-CDMC	C11H14ClNO	212.0837
2C-E	C12H19NO2	210.1489
2C-G	C12H19NO2	210.1489
2C-H	C10H15NO2	182.1176
2C-I	C10H14INO2	308.0142
2C-N	C10H14N2O4	227.1026
2-COOH-MDMB-PICA	C19H24N2O5	361.1758
2C-P	C13H21NO2	224.1645
2C-T-2	C12H19NO2S	242.1029
2C-T-7	C13H21NO2S	256.1336
2F-Deschloroketamine	C13H16FNO	222.1289
2-Fluoro-2-oxo PCE	C14H18FNO	236.1445
2'-Fluorofentanyl	C22H27FN2O	355.2180
2F-QMPSB	C22H20F2N2O4S	447.1185
2F-Viminol	C21H31FN2O	347.2493
2-Methyl alpha-PiHP	C17H25NO	260.2009
2-methyl AP-237	C18H26N2O	287.2118
2-Methyl-1,2,3,4-THBC	C12H14N2	187.1230
2-Methylmethcathinone (2-MMC)	C11H15NO	178.1226
2-Naphthyl U-47700	C20H26N2O	311.2118
3,4,5-Trimethoxyamphetamine	C12H19NO3	226.1438
3,4-Dichloro-alpha-PiHP	C16H21Cl2NO	314.1073
3,4-Difluoro-Isopropyl-U-47700	C18H26F2N2O	325.2086
3,4-Difluoro-N,N-Didesmethyl-U-47700	C14H18F2N2O	269.1460

© 2024 Kuai D et al. *JAMA Network Open.*

3,4-Difluoro-N-Desmethyl-U-47700	C15H20F2N2O	283.1617
3,4-Difluoro-U-47700	C16H22F2N2O	297.1773
3,4-Difluoro-U-48800	C17H24F2N2O	311.1930
3,4-Difluoro-U-49900	C18H26F2N2O	325.2086
3,4-Difluoro-U-50488	C19H26F2N2O	337.2086
3,4-Difluoro-U-51754	C17H24F2N2O	311.1930
3,4-Dimethyl Alpha-PVP	C17H25NO	260.2009
3,4-Dimethylmethcathione	C12H17NO	192.1383
3,4-Methylenedioxy PCP	C18H25NO2	288.1958
3,5-ADB-4en-PFUPPYCA	C21H27FN4O2	387.2191
3,5-Dimethyoxyphenol	C8H10O3	155.0703
3-Acetamidophenol	C8H9NO2	152.0706
3-CAF	C24H15FN2O2	383.1190
3-CDMC	C11H14ClNO	212.0837
3-Chlorocathinone	C9H10ClNO	184.0524
3-Chloromethcathinone	C10H12ClNO	198.0680
3Cl-PCP	C17H24ClN	278.167
3-Desoxy-MDPV	C17H23NO2	274.1802
3-fluoro-N-ethyl Hexedrone	C14H20FNO	238.1602
3F-MT-45	C24H31FN2	367.2544
3F-N-Ethylbuphedrone	C12H16FNO	210.1289
3F-PCP	C17H24FN	262.1966
3-HO-PCE	C14H21NO	220.1696
3-hydroxy Flubromazepam	C15H10BrFN2O2	348.9982
3-MeO-PCE	C15H23NO	234.1852
3-methoxy PCPy	C17H25NO	260.2009
3-Methyl Butyrylfentanyl	C24H32N2O	365.2587
3-Methyl fentanyl	C23H30N2O	351.2430
3-methyl PCP	C18H27N	258.2216
3-Methylmethcathinone (3-MMC)	C11H15NO	178.1226
3-Methylnordiazepam	C16H13ClN2O	285.0789
3-OH Phenazepam	C15H10BrClN2O2	364.9687
3-OH-PCP	C17H25NO	260.2009
4-(Trifluoromethyl) U-47700	C17H23F3N2O	329.1835
4-AcO-EPT	C17H24N2O2	289.1911
4-Aminoantipyrine	C11H13N3O	204.1131
4-ANBP	C18H22N2	267.1856
4-Anilino-1-Boc-Piperidine	C16H24N2O2	277.1911
4-ANPP	C19H24N2	281.2012
4Br-alpha-PVP	C15H20BrNO	310.0801
4-Bromomethcathinone	C10H12BrNO	242.0175
4-CDMC	C11H14ClNO	212.0837
4-CEC	C11H14ClNO	212.0837
4-Chloroamphetamine	C9H12ClN	170.0731
4'-Chlorodiazepam	C16H12Cl2N2O	319.0400
4-Chloromethamphetamine	C10H14ClN	184.0888
4Cl-alpha-PVP	C15H20ClNO	266.1306
4'Cl-Deschloroalprazolam	C17H13ClN4	309.0902
4Cl-Isopropylcathinone	C12H16ClNO	226.0993
4Cl-Pentedrone	C12H16ClNO	226.0993
4CN-AB-BICA	C19H24N4O2	341.1972
4CN-MMB-BINACA	C19H24N4O3	357.1921
4CN-MMB-BINACA 3-Methylbutanoic Acid	C18H22N4O3	343.1765
4-cyano CUMYL-BUT7AICA	C22H24N4O	361.2023
4-cyano CUMYL-BUTINACA	C22H24N4O	361.2023
4-cyano CUMYL-BUTINACA N-Butanoic Acid	C21H23N3O3	366.1812
4-cyano MDMB-BINACA	C20H26N4O3	371.2078
4-Ethylethcathinone	C13H19NO	206.1539
4-ethyl-n,n-Dimethylcathinone	C13H19NO	206.1539
4F-3-Methyl-alpha-PVP	C16H22FNO	264.1758
4F-AB-BINACA	C17H23FN4O2	335.1878
4F-ABINACA N-Butanoic Acid	C22H27N3O3	382.2125
4F-ABUTINACA	C22H28FN3O	370.2289
4F-alpha-PHP	C16H22FNO	264.1758
4F-alpha-PVP	C15H20FNO	250.1602
4F-CUMYL-5F-PINACA	C22H25F2N3O	386.2039
4F-Ethylphenidate	C15H20FNO2	266.1551
4F-MDMB-BICA	C20H27FN2O3	363.2079
4F-MDMB-BICA 3,3-Dimethylbutanoic Acid	C19H25FN2O3	349.1922

© 2024 Kuai D et al. *JAMA Network Open.*

4F-MDMB-BINACA	C19H26FN3O3	364.2031
4F-MDMB-BINACA 3,3-Dimethylbutanoic Acid	C18H24FN3O3	350.1875
4F-Methylphenidate	C14H18FNO2	252.1394
4-Formylaminoantipyrine	C12H13N3O2	232.1081
4F-Pentedrone	C12H16FNO	210.1289
4F-U-47931E	C15H21FN2O	265.1711
4-HO-DiPT	C16H24N2O	261.1961
4-HO-MET	C18H23NO2	219.1492
4'-hydroxy Nitazene	C20H24N4O3	369.1921
4-Hydroxy Xylazine	C12H16N2OS	237.1056
4-MeO-PCP	C18H27NO	274.2165
4-MeOPP	C11H16N2O	193.1335
4-Methoxybutyryl Fentanyl	C24H32N2O2	381.2172
4'-Methyl fentanyl	C23H30N2O	351.2430
4'-Methyl Hexedrone	C14H21NO	220.1696
4-methyl Pentedrone	C13H19NO	206.1539
4-Methylaminoantipyrine	C12H15N3O	218.1288
4-Methylaminorex	C10H12N2O	177.1022
4-Methyldiethcathinone	C14H21NO	220.1696
4-Methylethcathinone	C12H17NO	192.1383
4-Methylmethylphenidate	C15H21NO2	248.1645
4-Methyl-N-Ethyl Pentedrone	C14H21NO	220.1696
4OH-ADB-BINACA	C18H26N4O3	347.2078
4OH-MDMB-BICA	C20H28N2O4	361.2122
4OH-MDMB-BINACA	C19H27N3O4	362.2074
4-Phenyl Fentanyl	C28H32N2O	413.2587
4-Phenyl-U-51754	C23H30N2O	351.2430
5/6-APB	C11H13NO	176.1070
5-Aminoisotonitazene	C23H32N4O	381.2649
5Br-AKB-48	C23H30BrN3O	444.1645
5Br-THJ-018	C23H21BrN2O	421.0910
5Cl-AB-PINACA	C18H25ClN4O2	365.1739
5Cl-AKB-48	C23H30ClN3O	400.2150
5Cl-AMB-PICA	C20H27ClN2O3	379.1783
5Cl-MDMB-PICA	C21H29ClN2O3	393.194
5Cl-THJ-018	C23H21ClN2O	377.1415
5F JWH-018 Adamantyl Analogue	C24H30FNO	368.2384
5F-7-QUPAIC	C22H20FN3O2	378.1612
5F-AB-FUPPYCA	C20H26F2N4O2	393.2097
5F-ABICA	C19H26FN3O2	348.2082
5F-ADB	C20H28FN3O3	378.2188
5F-ADB 3,3-Dimethylbutanoic Acid	C19H26FN3O3	364.2031
5F-ADBICA	C20H28FN3O2	362.2238
5F-ADB-PINACA	C19H27FN4O2	363.2191
5F-AEB	C20H28FN3O3	378.2188
5F-AKB48 (5F-APINACA)	C23H30FN3O	384.2446
5F-AMB	C19H26FN3O3	364.2031
5F-AMB 3-Methylbutanoic Acid	C18H24FN3O3	350.1875
5F-APINAC	C23H29FN2O2	385.2286
5F-BEPIRAPIM	C25H30FN3O	408.2446
5F-BZO-POXIZID	C20H20FN3O2	354.1612
5F-CUMYL PINACA N-Pentanoic Acid	C22H25N3O3	380.1969
5F-CUMYL-P7AICA	C22H26FN3O	368.2132
5F-CUMYL-PeGACLONE	C25H27FN2O	391.2180
5F-CUMYL-PICA	C23H27FN2O	367.2180
5F-CUMYL-PINACA	C22H26FN3O	368.2133
5F-EDMB-PICA	C22H31FN2O3	391.2392
5F-EDMB-PINACA	C21H30FN3O3	392.2344
5F-EMB-PICA	C21H29FN2O3	377.2235
5-fluoro AB-PINACA	C18H25FN4O2	349.2034
5-fluoro CYPPICA	C18H23FN2O	303.1867
5-fluoro PY-PICA	C18H23FN2O	303.1867
5-Fluoropentyl-3-pyridinoylindole	C19H19FN2O	311.1554
5F-MDMB-P7AICA	C20H28FN3O3	378.2188
5F-MDMB-PICA	C21H29FN2O3	377.2235
5F-MDMB-PICA 3,3-Dimethylbutanoic Acid	C20H27FN2O3	363.2079
5F-MN-18	C23H22FN3O	376.1820
5F-MPP-PICA	C24H27FN2O3	411.2079
5F-NNEI	C24H23FN2O	375.1867

© 2024 Kuai D et al. *JAMA Network Open.*

5F-NPB-22	C22H20FN3O2	378.1612
5F-NPB-22 3-Carboxyindazole	C13H15FN2O2	251.1190
5F-PB-22 (5-fluoro QUPIC)	C23H21FN2O2	377.1660
5F-PB-22 3-Carboxyindole	C14H16FNO2	250.1238
5F-PCN	C23H22FN3O	376.1820
5F-PY-PINACA	C17H22FN3O	304.1820
5F-SDB-005	C23H21FN2O2	377.1660
5F-SDB-006	C21H23FN2O	339.1867
5F-THJ	C22H21FN4O	377.1772
5-IT	C11H14N2	175.1230
5-MAPB	C12H15NO	190.1226
5-MeO MiPT	C15H22N2O	247.1805
5-MeO NiPT	C14H20N2O	233.1648
5-MeO-Amt	C12H16N2O	205.1335
5-MeO-DALT	C17H22N2O	271.1805
5-MeO-DiPT	C17H26N2O	275.2118
5-MeO-DMT	C13H18N2O	219.1492
5-Methyl Etodesnitazene	C23H31N3O	366.2540
5OH-MDMB-PICA	C21H30N2O4	375.2278
5-Trifluoromethyl Isotonitazene	C24H30F3N3O	434.2414
6-Methoxy Methylone	C12H15NO4	238.1074
6-Monoacetylmorphine	C19H21NO4	328.1543
7-Amino Clonazepam	C15H12ClN3O	286.0742
7-Amino Flunitrazepam	C16H14FN3O	284.1194
7-Hydroxymitragynine	C23H30N2O5	415.2227
8-Aminoclonazolam	C17H14ClN5	324.1011
9(R)-Carboxy-HHC	C21H30O4	347.2217
9(R)-HHC	C21H32O2	317.2475
9(R)-HHC Acetate	C23H34O3	359.2581
9(R)-HHCH	C22H34O2	331.2632
9(R)-HHCP	C23H36O2	345.2788
9(S)-delta-6a,10a-THC	C21H30O2	315.2319
9(S)-delta-7-THC	C21H30O2	315.2319
9(S)-HHC	C21H32O2	317.2475
9-alpha-Hydroxy-HHC	C21H32O3	333.2424
9-beta-Hydroxy-HHC	C21H32O3	333.2424
A-796,260	C22H30N2O2	355.2380
A-834,735	C22H29NO2	340.2271
A-836339	C16H26N2O2S	311.1788
AB-001 (JWH-018 Adamantyl Analogue)	C24H31NO	350.2478
AB-005	C23H32N2O	353.2587
AB-BICA	C21H23N3O2	350.1863
AB-CHFUPYCA	C22H29FN4O2	401.2347
AB-CHMIATA	C22H31N3O2	370.2489
AB-CHMICA	C21H29N3O2	356.2333
AB-CHMINACA	C20H28N4O2	357.2285
AB-CHMINACA 2'-Indazole Isomer	C20H28N4O2	357.2285
AB-CHMINACA 3-Methylbutanoic Acid	C20H27N3O3	358.2125
AB-FUBICA	C21H22FN3O2	368.1769
AB-FUBINACA	C20H21FN4O2	369.1721
AB-FUBINACA Oxobutanoic Acid	C20H19FN4O4	399.1463
AB-FUBINACA-D4	C20H17[2H4]FN4O2	373.1972
ABO-4en-PINACA	C17H22N4O2	315.1816
AB-PINACA	C18H26N4O2	331.2129
AB-PINACA N-Pentanoic Acid	C18H24N4O4	361.1870
Acepromazine	C19H22N2OS	327.1526
Acetaminophen	C8H9NO2	152.0706
Acetyl Fentanyl	C21H26N2O	323.2118
Acetyl Fentanyl 4-Methylphenethyl Analog	C22H28N2O	337.2274
Acetylcodeine	C20H23NO4	342.1700
ACHMINACA	C25H33N3O	392.2696
Acrylfentanyl	C22H26N2O	335.2118
ADB-4en-PINACA	C19H26N4O2	343.2129
ADB-5'Br-4en-PINACA	C19H25BrN4O2	421.1234
ADB-5'Br-BINACA	C18H25BrN4O2	409.1234
ADB-5Br-INACA	C14H17BrN4O2	353.0608
ADB-5'Br-PINACA	C19H27BrN4O2	423.1390
ADB-BENZINACA	C21H24N4O2	365.1972
ADB-BICA	C22H25N3O2	364.2020

© 2024 Kuai D et al. *JAMA Network Open.*

ADB-BINAATA	C19H28N4O2	345.2285
ADB-BINACA	C18H26N4O2	331.2129
ADB-BINACA N-Butanoic Acid	C18H24N4O4	361.1870
ADB-FUBIATA	C23H26FN3O2	396.2082
ADB-FUBIATA 3,3-Dimethylbutanoic Acid	C23H25FN2O3	397.1922
ADB-FUBICA	C22H24FN3O2	382.1925
ADB-FUBINACA	C21H23FN4O2	383.1878
ADB-FUPYCA	C21H28F2N4O2	407.2253
ADB-HEXINACA	C20H30N4O2	359.2442
ADBICA	C20H29N3O2	344.2333
ADBICA N-Pentanoic Acid	C20H27N3O4	374.2074
ADBICA-D9	C20H20[2H9]N3O2	353.2897
ADB-INACA	C14H18N4O2	275.1503
ADB-PHETINACA	C22H26N4O2	379.2129
ADB-PINACA	C19H28N4O2	345.2285
ADB-PINACA N-Pentanoic Acid	C19H26N4O4	375.2027
ADB-PINACA-D9	C19H19[2H9]N4O2	354.2850
Adinazolam	C19H18ClN5	352.1324
AFUBIATA	C27H29FN2O	417.2337
AH-7921	C16H22Cl2N2O	329.1182
AH-8533	C16H23ClN2O	295.1572
AKB-48 (APINACA)	C23H31N3O	366.2540
AKB-48 N-Pentanoic Acid	C23H29N3O3	396.2282
ALD-52	C22H27N3O2	366.2176
Alfentanil	C21H32N6O3	417.2609
AL-LAD	C22H27N3O	350.2227
Allylescaline	C13H19NO3	238.1438
alpha-D2PV	C18H19NO	266.1539
alpha-Ethyl 2C-D	C13H21NO2	224.1645
alpha-Hydroxy Bromazolam	C17H13BrN4O	369.0346
alpha-Hydroxy Clonazolam	C17H12ClN5O3	370.0701
Alpha-Hydroxyalprazolam	C17H13ClN4O	325.0851
alpha'-Methyl Butyrylfentanyl	C24H32N2O	365.2587
alpha-Methyl Etonitazene	C23H30N4O3	411.2391
alpha-Methyltryptamine	C11H14N2	175.1230
Alpha-PBP	C14H19NO	218.1539
alpha-PCYP	C18H25NO	272.2009
Alpha-PHP	C16H23NO	246.1852
Alpha-PHpP (PV8)	C17H25NO	260.2009
alpha-PipBP	C15H21NO	232.1696
Alpha-PiHP	C16H23NO	246.1852
Alpha-PPP	C13H17NO	204.1383
Alpha-PVP	C15H21NO	232.1696
Alpha-PVT	C13H19NOS	238.1260
Alprazolam	C17H13ClN4	309.0902
Alprazolam-D5	C17H8[2H]5ClN4	314.1215
AM-1220	C26H26N2O	383.2118
AM-1235	C24H21FN2O3	405.1609
AM-1241	C22H22IN3O3	504.0779
AM-1248	C26H34N2O	391.2744
AM-2201	C24H22FNO	360.1758
AM-2201 8-Quinolinyl Carboxamide	C23H22FN3O	376.1820
AM-2201 Benzimidazole Analogue (FUBIMINA)	C23H21FN2O	361.1711
AM-2232	C24H20N2O	353.1648
AM-2233	C22H23IN2O	459.0928
AM-3102	C21H41NO2	340.3210
AM-630	C23H25IN2O3	505.0983
AM-679	C20H20INO	418.0662
AM-694	C20H19FINO	436.0568
AMB	C19H27N3O3	346.2125
a-Methyl fentanyl	C23H30N2O	351.2430
a-Methylacetyl Fentanyl	C22H28N2O	337.2274
Amidopyrine	C13H17N3O	232.1444
Amitraz	C19H23N3	294.1965
Amitriptyline	C20H23N	278.1903
Amoxapine	C17H16ClN3O	314.1055
Amphetamine	C9H13N	136.1121
Aniracetam	C12H13NO3	220.0968
AP-237	C17H24N2O	273.1961

© 2024 Kuai D et al. *JAMA Network Open.*

AP-238	C18H26N2O	287.2118
APICA	C24H32N2O	365.2587
APINAC (AKB57)	C23H30N2O2	367.2380
APP-BINACA	C21H24N4O2	365.1972
APP-BINACA 3-phenylpropanoic Acid	C21H23N3O3	366.1812
APP-CHMINACA	C24H28N4O2	405.2285
APP-FUBINACA	C24H21FN4O2	417.1721
APP-PICA	C23H27N3O2	378.2176
Aripiprazole	C23H27Cl2N3O2	448.1553
ATHPINACA	C24H31N3O2	394.2489
Atomoxetine	C17H21NO	256.1696
Atropine	C17H23NO3	290.1751
Azidoindolene 1 (1)	C21H28FN3O2	374.2238
Azidoindolene 1 (2)	C21H28FN3O2	374.2238
BB-22 (QUCHIC)	C25H24N2O2	385.1911
BB-22 3-Carboxyindole	C16H19NO2	258.1489
BBOP	C13H9NO2	212.0706
BDB	C11H15NO2	194.1176
Bentazepam	C17H16N2OS	297.1056
Benzocaine	C9H11NO2	166.0863
Benzodioxole Fentanyl	C27H28N2O3	429.2172
Benzoylecgonine	C16H19NO4	290.1387
Benztropine	C21H25NO	308.2009
Benzyl Carfentanil	C23H28N2O3	381.2172
Benzyl Fentanyl	C21H26N2O	323.2118
Benzyl Furanylfentanyl	C23H24N2O2	361.1911
Benzyl Phenylfentanyl	C25H26N2O	371.2118
Benzylone	C17H17NO3	284.1281
beta-Hydroxy Fentanyl	C22H28N2O2	353.2224
beta-Hydroxythiofentanyl	C20H26N2O2S	359.1788
Bipiperidinyl 4-ANPP	C24H33N3	364.2747
bk-EABDI	C15H21NO	232.1696
b-Methyl fentanyl	C23H30N2O	351.2430
b'-Phenyl Fentanyl	C28H32N2O	413.2587
Bretazenil	C19H20BrN3O3	418.0761
Bromadol	C22H28BrNO	402.1427
Bromantane	C16H20BrN	306.0852
Bromazepam	C14H10BrN3O	316.0080
Bromazolam	C17H13BrN4	353.0396
Bromo-Dragon FLY	C13H12BrNO2	294.0124
Brompheniramine	C16H19BrN2	319.0804
Brorphine	C20H22BrN3O	400.1019
Brotizolam	C15H10BrClN4S	392.9571
Bufotenine	C12H16N2O	205.1335
Buphedrone	C11H15NO	178.1226
Bupivacaine	C18H28N2O	289.2274
Buprenorphine	C29H41NO4	468.3108
Bupropion	C13H18ClNO	240.1150
Buspirone	C21H31N5O2	386.2551
Butonitazene	C24H32N4O3	425.2547
Butorphanol	C21H29NO2	328.2271
Butryl Fentanyl	C23H30N2O	351.2430
Butylone	C12H15NO3	222.1125
BZO-4en-POXIZID	C20H19N3O2	334.1550
BZO-CHMOXIZID	C22H23N3O2	362.1863
BZO-POXIZID	C20H21N3O2	336.1707
BZP	C11H16N2	177.1386
Caccure 907	C15H21NO2S	280.1366
Caffeine	C8H10N4O2	195.0877
Cannabigerol (CBG)	C21H32O2	317.2475
Carbamazepine	C15H12N2O	237.1022
Carbamazepine-10, 11 Epoxide	C15H12N2O2	253.0972
Carboxy-THC	C21H28O4	345.2060
Carfentanil	C24H30N2O3	395.2329
Carisoprodol	C12H24N2O4	261.1809
Cathinone	C9H11NO	150.0913
CB-13	C26H24O2	369.1849
CB-25	C25H41NO3	404.3159
CB-52	C26H43NO3	418.3316

© 2024 Kuai D et al. *JAMA Network Open.*

CB-86	C26H43NO3	418.3316
CBD	C21H30O2	315.2319
CBL-018	C24H23NO2	358.1802
CBN	C21H26O2	311.2006
Cephaeline	C28H38N2O4	467.2904
CH-FUBIATA	C23H25FN2O	365.2024
CH-HEXIATA	C22H32N2O	341.2587
Chlordiazepoxide	C16H14ClN3O	300.0898
Chloroquine	C18H26ClN3	320.1888
Chlorpheniramine	C16H19ClN2	275.1310
Chlorphine	C20H22ClN3O	356.1524
Chlorpromazine	C17H19ClN2S	319.1030
CHM-FUBIATA	C24H27FN2O	379.2180
CHO-4'Me-5'Br-FUBOXPYRA	C20H22BrFN2O2	421.0921
CH-PIATA	C21H30N2O	327.2431
CH-PIATA N-Pentanoic Acid	C21H28N2O3	357.2173
Ciclotizolam	C20H18BrClN4S	461.0197
Cinazepam	C19H14BrClN2O5	464.9847
Citalopram	C20H21FN2O	325.1711
Cl-2201	C24H21ClFNO	394.1369
Clobazam	C16H13ClN2O2	301.0738
Clomipramine	C19H23ClN2	315.1623
Clonazepam	C15H10ClN3O3	316.0484
Clonazolam	C17H12ClN5O2	354.0752
Clonidine	C9H9Cl2N3	230.0246
Cloniprazepam	C19H16ClN3O3	370.0953
Clonitazene	C20H23ClN4O2	387.1582
Clotizolam	C15H10Cl2N4S	349.0076
Clozapine	C18H19ClN4	327.1371
Cocaethylene	C18H23NO4	318.1700
Cocaine	C17H21NO4	304.1543
Codeine	C18H21NO3	300.1594
Coniine	C8H17N	128.1434
Cotinine	C10H12N2O	177.1022
CP-55,940	C24H40O3	377.3050
Crotonyl Fentanyl	C23H28N2O	349.2274
CUMYL-CBMICA	C23H26N2O	347.2118
CUMYL-INACA	C17H17N3O	280.1444
CUMYL-NBMICA	C26H30N2O	387.2431
CUMYL-PeGACLONE	C25H28N2O	373.2274
CUMYL-PICA	C23H28N2O	349.2274
CUMYL-PIPETINACA	C24H30N4O	391.2492
CUMYL-THPINACA	C23H27N3O2	378.2176
CUMYL-TsINACA	C24H23N3O3S	434.1533
Cyclobenzaprine	C20H21N	276.1747
Cyclobutylfentanyl	C24H30N2O	363.2431
Cyclohexyl Fentanyl	C26H34N2O	391.2744
Cyclopentyl Fentanyl	C25H32N2O	377.2587
Cyclopropaneacetylfentanyl	C24H30N2O	363.2431
Cyclopropyl Fentanyl	C23H28N2O	349.2274
Cyclopropyl Norfentanyl	C15H20N2O	245.1648
Cyclopropyl U-47700	C18H24Cl2N2O	355.1339
DBZP	C18H22N2	267.1856
Delorazepam	C15H10Cl2N2O	305.0243
Delta-4(8)-iso-THC	C21H30O2	315.2319
Delta-8 Carboxy-THC	C21H28O4	345.2060
Delta-8 THC	C21H30O2	315.2319
Delta-8-iso-THC	C21H30O2	315.2319
Delta-9-THC Acetate	C23H32O3	357.2424
Delta-9-THC Methyl Ether	C22H32O2	329.2475
Delta-9-THCB	C20H28O2	301.2162
Delta-9-THC-C8	C24H36O2	357.2788
Delta-9-THCE	C18H24O2	273.1849
Delta-9-THCH	C22H32O2	329.2475
Delta-9-THCO	C17H22O2	259.1693
Delta-9-THCP	C23H34O2	343.2632
Deoxymethoxetamine (DMXE)	C15H21NO	232.1696
Desalkylflurazepam	C15H10ClFN2O	289.0539
Desalkylgidazepam	C15H11BrN2O	315.0128

© 2024 Kuai D et al. *JAMA Network Open.*

Desalkylquazepam	C15H10ClFN2S	305.0310
Deschloroclotizolam	C15H11ClN4S	315.0466
Deschloroetizolam	C17H16N4S	309.1168
Deschloroketamine	C13H17NO	204.1383
Deschloronorketamine	C12H15NO	190.1226
Desipramine	C18H22N2	267.1856
Desmethylclomipramine	C18H21ClN2	301.1393
Desmethyldoxepin	C18H19NO	266.1539
Desmethylmoramide	C24H30N2O2	379.2380
Desmethylsertraline	C16H15Cl2N	292.0654
Desomorphine	C17H21NO2	272.1645
Despropionyl 2'-Fluoro ortho-Fluorofentanyl	C19H22F2N2	317.1824
Despropionyl 3-Methylfentanyl	C20H26N2	295.2169
Despropionyl Carfentanil	C21H26N2O2	339.2067
Despropionyl ortho-Fluorofentanyl	C19H23FN2	299.1918
Despropionyl ortho-Methylfentanyl	C20H26N2	295.2169
Despropionyl para-Chlorofentanyl	C19H23ClN2	315.1623
Detomidine	C12H14N2	187.1230
Dextro / Levo Methorphan	C18H25NO	272.2009
Dextrorphan / Levorphanol	C17H23NO	258.1852
Diacetylmorphine	C21H23NO5	370.1649
Diazepam	C16H13ClN2O	285.0789
Dibutylone	C13H17NO3	236.1281
Dichloroethcathinone	C11H13Cl2NO	246.0447
Dichloromethylphenidate	C14H17Cl2NO2	302.0709
Diclazepam	C16H12Cl2N2O	319.0399
Dicyclomine	C19H35NO2	310.2741
Didesmethylsibutramine	C15H22ClN	252.1514
Diethylone	C14H19NO3	250.1438
Diethylpentylone	C16H23NO3	278.1751
Difludiazepam	C16H11ClF2N2O	321.0601
Difluoro-cis-3-Methylfentanyl	C23H28F2N2O	387.2243
Difluorofentanyl	C22H26F2N2O	373.2086
Dihydrocodeine	C18H23NO3	302.1751
Diltiazem	C22H26N2O4S	415.1686
Dimethocaine	C16H26N2O2	279.2067
Dimethylamphetamine	C11H17N	164.1434
Dimethylone	C12H15NO3	222.1125
Dimethylpentylone	C14H19NO3	250.1438
Dimethyltryptamine	C12H16N2	189.1386
Diphenhydramine	C17H21NO	256.1696
Dipipanone	C24H31NO	350.2478
Dipyanone	C23H29NO	336.2322
DMPEA	C10H15NO2	182.1176
DOB	C11H16BrNO2	274.0437
DOM	C12H19NO2	210.1489
Donepezil	C24H29NO3	380.2220
Doxepin	C19H21NO	280.1696
Doxylamine	C17H22N2O	271.1805
Duloxetine	C18H19NOS	298.1260
EAM-2201	C26H26FNO	388.2071
Ecgonine Methyl Ester	C10H17NO3	200.1281
EDDP	C20H23N	278.1903
EDMB-PINACA	C21H31N3O3	374.2438
EG018	C28H25NO	392.2009
EG-2201	C28H24FNO	410.1915
EMB-FUBINACA	C22H24FN3O3	398.1875
EMDP	C19H21N	264.1747
Emetine	C29H40N2O4	481.3061
Ephedrine / Pseudoephedrine	C10H15NO	166.1226
Estazolam	C16H11ClN4	295.0745
Etaqualone	C17H16N2O	265.1335
Ethacathinone	C11H15NO	178.1226
ETH-LAD	C21H27N3O	338.2227
Ethoxyacetyl Fentanyl	C23H30N2O2	367.2380
Ethyl 4-ANPP	C21H28N2	309.2325
Ethylene Etonitazene	C23H30N4O3	411.2391
Ethylenedioxy-U-47700	C18H26N2O3	319.2016
Ethylenedioxy-U-51754	C19H28N2O3	333.2176

© 2024 Kuai D et al. *JAMA Network Open.*

Ethyleneoxynitazene	C22H26N4O3	395.2078
Ethylindole Fentanyl	C24H29N3O	376.2383
Ethylmorphine	C19H23NO3	314.1751
Ethylone	C12H15NO3	222.1125
Ethylphenidate	C15H21NO2	248.1645
Eticyclidine (PCE)	C14H21N	204.1747
Etilefrine	C10H15NO2	182.1176
Etizolam	C17H15ClN4S	343.0779
Etodesnitazene	C22H29N3O	352.2383
Etodolac	C17H21NO3	288.1594
Etoetonitazene	C24H32N4O4	441.2496
Etomidate	C14H16N2O2	245.1285
Etonitazene	C22H28N4O3	397.2234
Eutylone	C13H17NO3	236.1281
exo-THC	C21H30O2	315.2319
F-2201	C24H21F2NO	378.1664
FAB-144	C20H27FN2O	331.2180
FDU-NNEI	C26H19FN2O	395.1554
FDU-PB-22	C26H18FNO2	396.1394
Fenethylline	C18H23N5O2	342.1925
Fenfluramine	C12H16F3N	232.1308
Fentanyl	C22H28N2O	337.2274
Fentanyl Methyl Carbamate	C21H26N2O2	339.2067
Flecainide	C17H20F6N2O3	415.1451
Flephedrone	C10H12FNO	182.0976
Flualprazolam	C17H12ClFN4	327.0807
Flubromazepam	C15H10BrFN2O	333.0033
Flubromazolam	C17H12BrFN4	371.0302
Flubrotizolam	C15H10BrFN4S	376.9866
Fluclotizolam	C15H10ClFN4S	333.0372
Fluetizolam	C17H15FN4S	327.1074
Flunitazene	C20H23FN4O2	371.1878
Flunitrazepam	C16H12FN3O3	314.0936
Flunitrazolam	C17H12FN5O2	338.1048
Fluorexetamine (FXE)	C14H18FNO	236.1445
Fluoroamphetamine	C9H12FN	154.1027
Fluoroethamphetamine	C11H16FN	182.1340
Fluoroisobutyryl Fentanyl	C23H29FN2O	369.2337
Fluoro-JWH-019	C25H24FNO	374.1915
Fluoromethamphetamine	C10H14FN	168.1183
Fluorphine	C20H22FN3O	340.1820
Fluoxetine	C17H18F3NO	310.1413
Fluphenazine	C22H26F3N3OS	438.1822
Flurazepam	C21H23ClFN3O	388.1587
Flutoprazepam	C19H16ClFN2O	343.1008
Fluvoxamine	C15H21F3N2O2	319.1628
FUB-144	C23H24FNO	350.1915
FUB-AKB-48	C25H26FN3O	404.2133
FUBIMINA N-pentanoic acid	C23H20N2O3	373.1547
FUB-JWH-018	C26H18FNO	380.1445
FUB-NPB-22	C24H16FN3O2	398.1299
FUB-PB-22	C25H17FN2O2	397.1347
FUB-PB-22 3-Carboxyindole	C16H12FNO2	270.0925
Furanyl Fentanyl	C24H26N2O2	375.2067
Furanyl UF-17	C19H24N2O2	313.1911
Furanylethylfentanyl	C20H26N2O2	327.2067
Gabapentin	C9H17NO2	172.1332
Glimepiride	C24H34N4O5S	491.2323
Glipizide	C21H27N5O4S	446.1857
Glutethimide	C13H15NO2	218.1176
Guaifenesin	C10H14O4	199.0965
Haloperidol	C21H23ClFNO2	376.1474
Hexanoyl Fentanyl	C25H34N2O	379.2744
Hexedrone	C13H19NO	206.1539
Hexylone	C14H19NO3	250.1438
HU-210/HU-211	C25H38O3	387.2894
HU-308	C27H42O3	415.3207
HU-331	C21H28O3	329.2111
Hydrocodone	C18H21NO3	300.1594

© 2024 Kuai D et al. *JAMA Network Open.*

Hydromorphone	C17H19NO3	286.1438
Hydroxetamine (HXE)	C14H19NO2	234.1489
Hydroxybupropion	C13H18ClNO2	256.1099
Hydroxychloroquine	C18H26ClN3O	336.1837
Hydroxy-delta-8-THC	C21H30O3	331.2268
Hydroxyethylflurazepam	C17H14ClFN2O2	333.0801
Hydroxy-THC	C21H30O3	331.2268
Hydroxytriazolam	C17H12Cl2N4O	359.0461
Hydroxyzine	C21H27ClN2O2	375.1834
Iloperidone	C24H27FN2O4	427.2028
Imipramine	C19H24N2	281.2012
IMMA (BML-190)	C23H23ClN2O4	427.1419
Indole-3-acetic Acid (IAA)	C10H9NO2	176.0706
Indomethacin	C19H16ClNO4	358.0841
iso-Butonitazene	C24H32N4O3	425.2547
Isobutyl-PINAC	C17H24N2O2	289.1911
Isobutyryl Fentanyl	C23H30N2O	351.2430
Isopropylphenidate	C16H23NO2	262.1802
Isopropyl-U-47700	C18H26Cl2N2O	357.1495
Isotodesnitazene	C23H31N3O	366.2540
Isotonitazene	C23H30N4O3	411.2391
Itraconazole	C35H38Cl2N8O4	705.2466
JWH-007	C25H25NO	356.2009
JWH-011	C27H29NO	384.2322
JWH-015	C23H21NO	328.1696
JWH-016	C24H23NO	342.1852
JWH-018	C24H23NO	342.1852
JWH-018 6-Methoxyindole Analogue	C25H25NO2	372.1958
JWH-018 8-Quinolinyl Carboxamide	C23H23N3O	358.1914
JWH-018 Benzimidazole Analogue	C23H22N2O	343.1805
JWH-018 N-(1,1-Dimethylpropyl) Isomer	C24H23NO	342.1852
JWH-018 N-(4,5-Epoxypentyl) Analogue	C24H21NO2	356.1645
JWH-018 N-(5-Bromopentyl) Analogue	C24H22BrNO	420.0958
JWH-018 N-(5-Chloropentyl) Analogue	C24H22ClNO	376.1463
JWH-018 N-Pentanoic Acid	C24H21NO3	372.1594
JWH-018-D9	C24H14[2H9]NO	351.2417
JWH-019	C25H25NO	356.2009
JWH-020	C26H27NO	370.2165
JWH-022	C24H21NO	340.1696
JWH-030	C20H21NO	292.1696
JWH-031	C21H23NO	306.1852
JWH-071	C21H17NO	300.1383
JWH-072	C22H19NO	314.1539
JWH-073	C23H21NO	328.1696
JWH-073 2-Methylnaphthyl Analogue	C24H23NO	342.1852
JWH-073 6-Methoxyindole Analogue	C24H23NO2	358.1802
JWH-080	C24H23NO2	358.1802
JWH-081	C25H25NO2	372.1958
JWH-081 N-(Cyclohexylmethyl) Analogue	C27H27NO2	398.2115
JWH-098	C26H27NO2	386.2115
JWH-116	C26H27NO	370.2165
JWH-122	C25H25NO	356.2009
JWH-122 N-(4-Pentenyl) Analogue	C25H23NO	354.1852
JWH-133	C22H32O	313.2526
JWH-145	C26H25NO	368.2009
JWH-146	C28H29NO	396.2322
JWH-147	C27H27NO	382.2165
JWH-149	C26H27NO	370.2165
JWH-167	C21H23NO	306.1852
JWH-175	C24H25N	328.2060
JWH-176	C25H24	325.1951
JWH-180	C25H25NO	356.2009
JWH-182	C27H29NO	384.2322
JWH-193	C26H26N2O2	399.2067
JWH-198	C26H26N2O3	415.2016
JWH-200	C25H24N2O2	385.1911
JWH-200 Analogue	C22H30N2O2	355.2380
JWH-201	C22H25NO2	336.1958
JWH-203	C21H22ClNO	340.1475

© 2024 Kuai D et al. *JAMA Network Open.*

JWH-210	C26H27NO	370.2165
JWH-213	C27H29NO	384.2322
JWH-249	C21H22BrNO	384.0958
JWH-250	C22H25NO2	336.1958
JWH-251	C22H25NO	320.2009
JWH-302	C22H25NO2	336.1958
JWH-307	C26H24FNO	386.1915
JWH-309	C30H27NO	418.2165
JWH-368	C26H24FNO	386.1915
JWH-369	C26H24ClNO	402.1619
JWH-370	C27H27NO	382.2165
JWH-387	C24H22BrNO	420.0958
JWH-398	C24H22ClNO	376.1463
JWH-412	C24H22FNO	360.1758
JWH-424	C24H22BrNO	420.0958
Kavain	C14H14O3	231.1016
Ketamine	C13H16ClNO	238.0993
Ketamine Hydroxylimine Precursor	C13H16ClNO	238.0993
Ketoconazole	C26H28Cl2N4O4	531.1560
Ketoprofen	C16H14O3	255.1016
KM 233	C25H30O2	363.2319
Lacosamide	C13H15FN2O2	251.1390
Lamotrigine	C9H7N5Cl2	256.0151
Laudanosine	C21H27NO4	358.2013
Levamisole	C11H12N2S	205.0794
Levetiracetam	C8H14N2O2	171.1128
Lidocaine	C14H22N2O	235.1805
Lisdexamphetamine	C15H25N3O	264.2070
Loperamide	C29H33ClN2O2	477.2303
Lorazepam	C15H10Cl2N2O2	321.0192
Lormetazepam	C16H12Cl2N2O2	335.0349
Loxapine	C18H18ClN3O	328.1211
LSD	C20H25N3O	324.2070
M-144	C22H30FNO	344.2384
MAB-CHMINACA	C21H30N4O2	371.2442
MAB-CHMINACA 3,3-Dimethylbutanoic Acid	C21H29N3O3	372.2282
MA-CHMINACA	C21H29N3O3	372.2282
MAM-2201	C25H24FNO	374.1915
MAM-2201 N-(5-Chloropentyl) Analogue	C25H24ClNO	390.1619
Maprotiline	C20H23N	278.1903
MBDB	C12H17NO2	208.1332
MBZP	C12H18N2	191.1543
MCHB-1	C28H37N3O2	448.2959
mCPP	C10H13ClN2	197.0840
MDA	C10H13NO2	180.1019
MDA 19	C21H23N3O2	350.1863
MDA 77	C21H23N3O3	366.1812
MDAI	C10H11NO2	178.0863
MDDMA	C12H17NO2	208.1332
MDEA	C12H17NO2	208.1332
MDMA	C11H15NO2	194.1176
MDMA-D5	C11H10[2H]5NO2	199.1489
MDMB-3en-BINACA	C19H25N3O3	344.1969
MDMB-4en-PICA	C21H28N2O3	357.2173
MDMB-4en-PINACA	C20H27N3O3	358.2125
MDMB-4en-PINACA 3,3-Dimethylbutanoic Acid	C19H25N3O3	344.1969
MDMB-5'Br-BINACA	C19H26BrN3O3	424.1230
MDMB-5Br-INACA	C15H18BrN3O3	368.0604
MDMB-5Me-INACA	C16H21N3O3	304.1656
MDMB-BENZICA	C23H26N2O3	379.2016
MDMB-BICA	C20H28N2O3	345.2173
MDMB-BINACA	C19H27N3O3	346.2125
MDMB-BUTINACA 3,3-Dimethylbutanoic Acid	C18H25N3O3	332.1969
MDMB-CHM7AICA	C22H31N3O3	386.2438
MDMB-CHMCZCA	C27H34N2O3	435.2642
MDMB-CHMICA	C23H32N2O3	385.2486
MDMB-CHMINACA	C22H31N3O3	386.2438
MDMB-FUBICA	C23H25FN2O3	397.1922
MDMB-FUBICA 3,3-Dimethylbutanoic Acid	C22H23FN2O3	383.1766

MDMB-FUBINACA	C22H24FN3O3	398.1875
MDMB-FUBINACA 3,3-Dimethylbutanoic Acid	C21H22FN3O3	384.1718
MDMB-ICA	C16H20N2O3	289.1547
MDMB-INACA	C15H19N3O3	290.1499
MDMB-MINACA	C16H21N3O3	304.1656
MDMB-PICA	C21H30N2O3	359.2329
MDP2P	C10H10O3	179.0703
MDPBP	C15H19NO3	262.1438
MDPPP	C14H17NO3	248.1281
MDPV	C16H21NO3	276.1594
Mebroqualone	C15H11BrN2O	315.0128
Meclonazepam	C16H12ClN3O3	330.064
Medazepam	C16H15ClN2	271.0997
Medetomidine	C13H16N2	201.1386
Meloxicam	C14H13N3O4S2	352.0420
Memantine	C12H21N	180.1747
Menitazene	C21H26N4O2	367.2129
MeO-MDA	C11H15NO3	210.1125
Meperidine	C15H21NO2	248.1645
Mephedrone	C11H15NO	178.1226
Mepirapim	C19H27N3O	314.2227
Mepivacaine	C15H22N2O	247.1805
MePPP	C14H19NO	218.1539
Meprobamate	C9H18N2O4	219.1339
Meptazinol	C15H23NO	234.1852
Mescaline	C11H17NO3	212.1281
Mesoridazine	C21H26N2OS2	387.1559
meta-Chlorofentanyl	C22H27ClN2O	371.1885
meta-Fluorofentanyl	C22H27FN2O	355.2180
Metaxalone	C12H15NO3	222.1125
Methacrylfentanyl	C23H28N2O	349.2274
Methadone	C21H27NO	310.2165
Methamphetamine	C10H15N	150.1277
Methaqualone	C16H14N2O	251.1179
Methcathinone	C10H13NO	164.1070
Methedrone	C11H15NO2	194.1176
Methionitazene	C21H26N4O2S	399.1849
Methiopropamine	C8H13NS	156.0842
Methocarbamol	C11H15NO5	242.1023
Methohexital	C14H18N2O3	263.1390
Methoxetamine	C15H21NO2	248.1645
Methoxy U-47700	C16H22Cl2N2O2	345.1131
Methoxyacetyl Fentanyl	C22H28N2O2	353.2224
Methoxyamphetamine	C10H15NO	166.1226
Methoxymethamphetamine	C11H17NO	180.1383
Methylenedioxy-alpha-PHP	C17H23NO3	290.1751
Methylenedioxy-alpha-PiHP	C17H23NO3	290.1751
Methylenedioxynitazene	C21H24N4O4	397.1870
Methylenedioxy-PV8	C18H25NO3	304.1907
Methylenedioxy-U-47700	C17H24N2O3	305.1860
Methylhexanamine (DMAA 1)	C7H17N	116.1434
Methylhexanamine (DMAA 2)	C7H17N	116.1434
Methylone	C11H13NO3	208.0968
Methylone-D3	C11H10[2H]3NO3	211.1157
Methylphenidate	C14H19NO2	234.1489
Metizolam	C16H13ClN4S	329.0622
Metoclopramide	C14H22ClN3O2	300.1473
Metodesnitazene	C21H27N3O	338.2227
Metonitazene	C21H26N4O3	383.2078
Mexiletine	C11H17NO	180.1383
MFUBINAC	C16H13FN2O2	285.1034
Midazolam	C18H13ClFN3	326.0855
Mirtazapine	C17H19N3	266.1652
Mitragynine	C23H30N2O4	399.2278
MMB-2201	C20H27FN2O3	363.2079
MMB-4en-PICA	C20H26N2O3	343.2016
MMB-4en-PINACA	C19H25N3O3	344.1969
MMB-5Br-INACA	C14H16BrN3O3	354.0448
MMB-CHMICA	C22H30N2O3	371.2329

© 2024 Kuai D et al. *JAMA Network Open.*

MMB-FUBGACLONE	C24H23FN2O3	407.1766
MMB-FUBICA	C22H23FN2O3	383.1766
MMB-FUBINACA	C21H22FN3O3	384.1718
MMB-FUBINACA 3-Methylbutanoic Acid	C20H20FN3O3	370.1562
MMB-ICA	C15H18N2O3	275.1390
MMB-PICA	C20H28N2O3	345.2173
MN-18	C23H23N3O	358.1914
MN-25	C26H37N3O3	440.2912
MN-25 2-Methyl Derivative	C27H39N3O3	454.3064
MO-CHMINACA	C22H30N2O4	387.2278
Modafinil	C15H15NO2S	274.0896
Monoethylglycinexylidide	C12H18N2O	207.1492
MOPPP	C14H19NO2	234.1489
Morphine	C17H19NO3	286.1438
Morphine-D3	C17H16[2H]3NO3	289.1626
MPBP	C15H21NO	232.1695
MPHP	C17H25NO	260.2009
MT-45	C24H32N2	349.2638
MXPr	C16H23NO2	262.1802
N-(2C-B) Fentanyl	C24H31BrN2O3	475.1591
N-(2C-E) Fentanyl	C26H36N2O3	425.2799
N-(2C-I) Fentanyl	C24H31IN2O3	523.1452
N-(2C-N) Fentanyl	C24H31N3O5	442.2337
N-(2C-P) Fentanyl	C27H38N2O3	439.2955
N-(DOBU) Fentanyl	C29H42N2O3	467.3268
N-(DOM) Fentanyl	C26H36N2O3	425.2799
N,N-Didesmethyl Loperamide	C27H29ClN2O2	449.1990
N,N-Didesmethyl U-47700	C14H18Cl2N2O	301.0869
N,N-Diethyltryptamine	C14H20N2	217.1699
N,N-Dimethylphenethylamine	C10H15N	150.1277
N,N-DMT N-Oxide	C12H16N2O	205.1335
N-acetyl 25I-NBOMe	C20H24INO4	470.0823
Nalbuphine	C21H27NO4	358.2013
Naloxone	C19H21NO4	328.1543
Naltrexone	C20H23NO4	342.1700
Naphyrone	C19H23NO	282.1852
Naproxen	C14H14O3	231.1016
N-benzyl-3,4-DMA	C18H23NO2	286.1802
N-Boc Norfentanyl	C19H28N2O3	333.2173
N-butyl Buphedrone	C14H21NO	220.1696
N-butyl Butylone	C15H21NO3	264.1594
N-butyl Hexedrone	C16H25NO	248.2009
N-Butyl N-Methyl Butylone	C16H23NO3	278.1751
N-butyl Pentylone	C16H23NO3	278.1751
N-Cyclohexyl Butylone	C17H23NO3	290.1751
N-Cyclohexyl Methylone	C16H21NO3	276.1594
N-desethyl Etonitazene	C20H24N4O3	369.1921
N-Desethyl Isotonitazene	C21H26N4O3	383.2078
N-Desethyl Metonitazene	C19H22N4O3	355.1765
N-Desethyl Protonitazene	C21H26N4O3	383.2078
N-desmethyl Loperamide	C28H31ClN2O2	463.2148
N-Desmethyl Rilmazolam	C18H13Cl2N5O	386.0570
N-Desmethyl U-47700	C15H20Cl2N2O	315.1026
NE-CHMIMO	C26H25NO	368.2009
N-Ethyl 2C-B	C12H18BrNO2	288.0594
N-ethyl Deschloroketamine	C14H19NO	218.1539
N-ethyl Heptedrone	C15H23NO	234.1852
N-Ethyl Hexedrone	C14H21NO	220.1696
N-ethyl Hexylone	C15H21NO3	264.1594
N-ethyl Pentedrone	C13H19NO	206.1539
N-Ethyl Pentylone	C14H19NO3	250.1438
N-ethyl Phenethylamine	C10H15N	150.1277
N-Ethylamphetamine	C11H17N	164.1434
N-Ethylbuphedrone	C12H17NO	192.1383
N-Ethyl-N-Methyl Butylone	C14H19NO3	250.1438
N-ethyl-U-477700	C17H24Cl2N2O	343.1339
N-formyl 4-ANPP	C20H24N2O	309.1961
Nicotine	C10H14N2	163.1230
Nifedipine	C17H18N2O6	347.1238

© 2024 Kuai D et al. *JAMA Network Open.*

Nifoxipam	C15H10FN3O4	316.0728
Nimetazepam	C16H13N3O3	296.1029
N-Isopropyl Butylone	C14H19NO3	250.1438
Nitazene	C20H24N4O2	353.1972
Nitazene Dihydrofuran Variant	C22H26N4O3	395.2078
Nitemazepam	C16H13N3O4	312.0979
Nitrazolam	C17H13N5O2	320.1142
NM-2201	C24H22FNO2	376.1707
NMDMSB	C20H19NO4S	370.1108
N-methyl Carfentanyl	C17H24N2O3	305.1860
N-Methyl Cyclazodone	C13H14N2O2	231.1128
N-methyl Cyclopropyl Norfentanyl	C16H22N2O	259.1805
N-methyl Ethylone	C13H17NO3	236.1281
N-methyl N-cyclohexyl Methylone	C17H23NO3	290.1751
N-methyl Norfentanyl	C15H22N2O	247.1805
N-methyl N-propyl Methylone	C14H19NO3	250.1438
N-methyl para-Methylphenyl Norfentanyl	C20H24N2O	309.1961
N-methyl U-47931E	C16H23BrN2O	339.1067
N-methyl-2-AI	C10H13N	148.1121
N-Methyltryptamine	C11H14N2	175.1230
NNEI	C24H24N2O	357.1961
Norbuprenorphine	C25H35NO4	414.2639
Norcarfentanil	C16H22N2O3	291.1703
Norclozapine	C17H17ClN4	313.1215
Norcocaine	C16H19NO4	290.1387
Norcodeine	C17H19NO3	286.1438
Nordiazepam	C15H11ClON2	271.0633
Norfentanyl	C14H20N2O	233.1648
Norflunitrazepam	C15H10FN3O3	300.0779
Norfluoxetine	C16H16F3NO	296.1257
Norfuranylfentanyl	C16H18N2O2	271.1441
Norketamine	C12H14ClNO	224.0837
Normeperidine	C14H19NO2	234.1489
Noroxycodone	C17H19NO4	302.1387
Norpropoxyphene	C21H27NO2	326.2115
Norpseudoephedrine / Phenylpropanolamine	C9H13NO	152.1070
Nortriptyline	C19H21N	264.1747
Noscapine	C22H23NO7	414.1547
NPB-22	C22H21N3O2	360.1707
N-Phenethyl Oxymorphone	C24H25NO4	392.1856
N-Phenethyl-N-Phenylpropionamide	C17H19NO	254.1539
N-Piperidinyl 4'-hydroxy Nitazene	C21H24N4O3	381.1921
N-Piperidinyl Etonitazene	C23H28N4O3	409.2234
N-Piperidinyl Heptylone	C19H27NO3	318.2064
N-Piperidinyl Isotonitazene	C24H30N4O3	423.2391
NPP	C13H17NO	204.1383
N-propionyl Norfentanyl	C17H24N2O2	289.1911
N-propyl Buphedrone	C13H19NO	206.1539
N-propyl Butylone	C14H19NO3	250.1438
N-Propyl N-Methyl Butylone	C15H21NO3	264.1594
N-propyl Pentedrone	C14H21NO	220.1696
N-Propylamphetamine	C12H19N	178.1590
N-Pyrrolidino 4'-hydroxy Nitazene	C20H22N4O3	367.1765
N-Pyrrolidino Etodesnitazene	C22H27N3O	350.2227
N-Pyrrolidino Etonitazene	C22H26N4O3	395.2078
N-Pyrrolidino Isotonitazene	C23H28N4O3	409.2234
N-Pyrrolidino Metodesnitazene	C21H25N3O	336.2070
N-Pyrrolidino Metonitazene	C21H24N4O3	381.1921
N-Pyrrolidino Protonitazene	C23H28N4O3	409.2234
O-AMKD	C16H21NO3	276.1594
Ocfentanil	C22H27FN2O2	371.2129
O-Desmethyltramadol	C15H23NO2	250.1802
O-Desmethylvenlafaxine	C16H25NO2	264.1958
Olanzapine	C17H20N4S	313.1482
Oliceridine	C22H30N2O2S	387.2101
ORG 28611	C23H33N3O2	384.2646
Orphenadrine	C18H23NO	270.1852
Orphine	C20H23N3O	322.1914
ortho-chlorofentanyl	C22H27ClN2O	371.1885

© 2024 Kuai D et al. *JAMA Network Open.*

ortho-Fluoro Furanylfentanyl	C24H25FN2O2	393.1973
ortho-Fluorofentanyl	C22H27FN2O	355.2180
ortho-Isopropyl Furanylfentanyl	C27H32N2O2	417.2537
ortho-Methoxy Furanylfentanyl	C25H28N2O3	405.2173
ortho-Methyl Acrylfentanyl	C23H28N2O	349.2274
ortho-Methyl Fentanyl	C23H30N2O	351.2430
ortho-Methyl Furanylfentanyl	C25H28N2O2	389.2224
ortho-Methyl Methoxyfentanyl	C23H30N2O2	367.2380
ortho-Methylacetyl Fentanyl	C22H28N2O	337.2274
Oxazepam	C15H11ClN2O2	287.0582
Oxycodone	C18H21NO4	316.1543
Oxymorphone	C17H19NO4	302.1387
Papaverine	C20H21NO4	340.1543
para-Bromo 4-ANPP	C19H23BrN2	359.1117
para-Bromofentanyl	C22H27BrN2O	415.1380
para-Chloro Acetyfentanyl	C21H25ClN2O	357.1728
para-Chloro Acrylfentanyl	C22H25ClN2O	369.1728
para-Chloro Cyclopentylfentanyl	C25H31ClN2O	411.2198
para-Chloro Cyclopropylfentanyl	C23H27ClN2O	383.1885
para-Chloro Furanylfentanyl	C24H25ClN2O2	409.1677
para-Chloro Valerylfentanyl	C24H31ClN2O	399.2198
para-Chlorofentanyl	C22H27ClN2O	371.1885
para-Chloroisobutyryl Fentanyl	C23H29ClN2O	385.2041
Para-Fluoro 4-ANBP	C18H21FN2	285.1762
para-Fluoro 4-Anilino-1-Boc-Piperidine	C16H23FN2O2	295.1816
para-Fluoro Acetylfentanyl	C21H25FN2O	341.2024
para-Fluoro Cyclopropylfentanyl	C23H27FN2O	367.2180
para-Fluoro Phenethyl 4-ANPP	C27H31FN2	403.2544
para-Fluoro Valerylfentanyl	C24H31FN2O	383.2493
para-Fluoroacryl Fentanyl	C22H25FN2O	353.2024
para-Fluorobutyryl Fentanyl	C23H29FN2O	369.2337
para-Fluorocyclopropylbenzylfentanyl	C22H25FN2O	353.2024
para-Fluorofentanyl	C22H27FN2O	355.2180
para-Methoxy 4-ANPP	C20H26N2O	311.2118
para-Methoxy Acrylfentanyl	C23H28N2O2	365.2224
para-Methoxy Fentanyl	C23H30N2O2	367.2380
para-Methoxy Methoxyacetylfentanyl	C23H30N2O3	383.2329
para-Methoxyacetyl Fentanyl	C22H28N2O2	353.2224
para-Methyl Acrylfentanyl	C23H28N2O	349.2274
para-Methyl AP-237	C18H26N2O	287.2118
para-Methyl Cyclopropylfentanyl	C24H30N2O	363.2431
para-Methyl Fentanyl	C23H30N2O	351.2430
para-Methyl Isobutyrylfentanyl	C24H32N2O	365.2587
para-Methyl Tetrahydrofuranylfentanyl	C25H32N2O2	393.2537
para-Methylacetyl Fentanyl	C22H28N2O	337.2274
para-Toluoylfentanyl	C27H30N2O	399.2431
Paroxetine	C19H20FNO3	330.1500
PB-22	C23H22N2O2	359.1754
PB-22 3-Carboxyindole	C14H17NO2	232.1332
Pentazocine	C19H27NO	286.2165
Pentedrone	C12H17NO	192.1383
Pentylone	C13H17NO3	236.1281
Perphenazine	C21H26ClN3OS	404.1558
PF-03550096	C19H28N4O4	377.2183
Phenacetin	C10H13NO2	180.1019
Phenazepam	C15H10BrClN2O	348.9738
Phenazolam	C17H12BrClN4	387.0007
Phencyclidine (PCP)	C17H25N	244.2060
Phendimetrazine	C12H17NO	192.1383
Phenethyl-4-ANPP	C27H32N2	385.2638
Phenibut	C10H13NO2	180.1019
Pheniramine	C16H20N2	241.1699
Phenmetrazine	C11H15NO	178.1226
Phenpromethamine	C10H15N	150.1277
Phensuximide	C11H11NO2	190.0863
Phentermine	C10H15N	150.1277
Phenyl Fentanyl	C26H28N2O	385.2274
Phenylacetyl Fentanyl	C27H30N2O	399.2431
Phenylbutazone	C19H20N2O2	309.1598

© 2024 Kuai D et al. *JAMA Network Open.*

Phenyltoloxamine	C17H21NO	256.1696
Phenytoin	C15H12N2O2	253.0972
Piperidylthiambutene	C17H21NS2	304.1188
Pivaloylfentanyl	C24H32N2O	365.2587
PMMA	C11H17NO	180.1383
Pramiracetam	C14H27N3O2	270.2176
Pravadoline (WIN-48,098)	C23H26N2O3	379.2016
Primidone	C12H14N2O2	219.1128
Procainamide	C13H21N3O	236.1757
Prochlorperazine	C20H24ClN3S	374.1452
Promazine	C17H20N2S	285.1420
Promethazine	C17H20N2S	285.1420
Propoxyphene	C22H29NO2	340.2271
Propylhexedrine	C10H21N	156.1747
Propylnitazene	C23H30N4O2	395.2442
Propylone	C13H17NO3	236.1281
Propyl-U-47700	C18H26Cl2N2O	357.1495
Protodesnitazene	C23H31N3O	366.2540
Protonitazene	C23H30N4O3	411.2391
Protriptyline	C19H21N	264.1747
PSB-SB1202	C23H26O4	367.1904
Psilocin	C12H16N2O	205.1335
Psilocybin	C12H17N2O4P	285.0999
PTI-1	C21H29N3S	356.2155
PTI-2	C23H33N3OS	400.2417
PX1	C23H26FN3O2	396.2082
PX2	C22H25FN4O2	397.2034
Pyrazolam	C16H12BrN5	354.0348
Pyrilamine	C17H23N3O	286.1914
Pyrovalerone	C16H23NO	246.1852
Pyrrolidino Variant Etonitazene	C23H28N4O3	409.2234
Quetiapine	C21H25N3O2S	384.1740
Quinidine	C20H24N2O2	325.1911
Quinine	C20H24N2O2	325.1911
R-6890	C21H24ClN3O	370.1681
Ramelteon	C16H21NO2	260.1645
RCS-4	C21H23NO2	322.1802
RCS-4 C4 Homolog	C20H21NO2	308.1645
RCS-8	C25H29NO2	376.2271
Remifentanyl Acid	C18H26N2O5	363.1914
Rilmazafone	C21H20Cl2N6O3	475.1047
Rilmazolam	C19H15Cl2N5O	400.0726
Risperidone	C23H27FN4O2	411.2191
Rolicyclidine	C16H23N	230.1903
Ropivacaine	C17H26N2O	275.2118
Salvinorin A	C23H28O8	433.1857
Salvinorin B	C21H26O7	391.1751
SBD-006	C21H24N2O	321.1961
Scopolamine	C17H21NO4	304.1543
SDB-005	C23H22N2O2	359.1754
SDB-006 N-Phenyl Analogue	C20H22N2O	307.1805
sec-Butonitazene	C24H32N4O3	425.2547
Senecioyl Fentanyl	C24H30N2O	363.2431
SER-601	C28H38N2O2	435.3006
Sertraline	C17H17Cl2N	306.0811
Sibutramine	C17H26ClN	280.1827
Sildenafil	C22H30N6O4S	475.2122
Strychnine	C21H22N2O2	335.1754
STS-135	C24H31FN2O	383.2493
Sufentanil	C22H30N2O2S	387.2101
Suvorexant	C23H23ClN6O2	451.1644
Tadalafil	C22H19N3O4	390.1448
Tamsulosin	C20H28N2O5S	409.1792
Tapentadol	C14H23NO	222.1852
Temazepam	C16H13ClN2O2	301.0738
Tenocyclidine	C15H23NS	250.1624
Tertylone	C14H19NO3	250.1438
Tetrahydrofuran Fentanyl	C24H30N2O2	379.2380
Tetrahydrothiophene Fentanyl	C24H30N2OS	395.2152

© 2024 Kuai D et al. *JAMA Network Open.*

Tetrahydrozoline	C13H16N2	201.1386
Tetramethylcyclopropyl Fentanyl	C27H36N2O	405.2900
TFMPP	C11H13F3N2	231.1104
THC	C21H30O2	315.2319
THCA	C22H30O4	359.2217
THCA-A	C22H30O4	359.2217
THCVA-A	C20H26O4	331.1904
Thebaine	C19H21NO3	312.1594
Theophylline	C7H8N4O2	181.0720
Thienyl Fentanyl	C19H24N2OS	329.1682
Thiofentanyl	C20H26N2OS	343.1838
Thionordiazepam	C15H11ClN2S	287.0404
Thiophene Fentanyl	C24H26N2OS	391.1839
Thioridazine	C21H26N2S2	371.1610
THJ	C22H22N4O	359.1866
THJ-018	C23H22N2O	343.1805
THJ-2201	C23H21FN2O	361.1711
Tianeptine	C21H25ClN2O4S	437.1296
Ticlopidine	C14H14ClNS	264.0608
Tiletamine	C12H17NOS	224.1104
Tizanidine	C9H8ClN5S	254.0262
Tofisopam	C22H26N2O4	383.1965
Topiramate	C12H21NO8S	340.1061
Tramadol	C16H25NO2	264.1958
Tranylcypromine	C9H11N	134.0964
Trazodone	C19H22ClN5O	372.1586
Triazolam	C17H12Cl2N4	343.0512
Trifluoperazine	C21H24F3N3S	408.1716
Trihexyphenidyl	C20H31NO	302.2478
Trimipramine	C20H26N2	295.2169
Triprolidine	C19H22N2	279.1856
Tryptamine	C10H12N2	161.1073
U-47700	C16H22Cl2N2O	329.1182
U-47931E	C15H21BrN2O	325.0910
U-48520	C16H23ClN2O	295.1572
U-48800	C17H24Cl2N2O	343.1338
U-49900	C18H26N2OCl2	357.1495
U-50488	C19H26Cl2N2O	369.1495
U-51754	C17H24Cl2N2O	343.1338
U-62066	C22H30Cl2N2O2	425.1757
U-69593	C22H32N2O2	357.2537
UF-17	C17H26N2O	275.2118
UR-144	C21H29NO	312.2322
UR-144 N-(5-Bromopentyl) Analogue	C21H28BrNO	390.1427
UR-144 N-(5-Chloropentyl) Analogue	C21H28ClNO	346.1932
UR-144 N-Heptyl Analogue	C23H33NO	340.2635
UR-144 N-Pentanoic Acid	C21H27NO3	342.2064
URB-447	C25H21ClN2O	401.1415
Urea Fentanyl	C22H29N3O	352.2383
Valerylfentanyl	C24H32N2O	365.2587
Vardenafil	C23H32N6O4S	489.2279
Varenicline	C13H13N3	212.1182
Venlafaxine	C17H27NO2	278.2115
Verapamil	C27H38N2O4	455.2904
Voriconazole	C16H14F3N5O	350.1223
W15	C19H21ClN2O2S	377.1085
W18	C19H20ClN3O4S	422.0935
Warfarin	C19H16O4	309.1121
WIN 55,212-3	C27H26N2O3	427.2016
WIN-54,461	C23H25BrN2O3	457.1121
XLR-11	C21H28FNO	330.2228
XLR-11 N-(4-Pentenyl) Analogue	C21H27NO	310.2165
XLR-11-D5	C21H23[2H5]FNO	335.2542
XLR-12	C20H24F3NO	352.1883
Xylazine	C12H16N2S	221.1107
Yohimbine	C21H26N2O3	355.2016
Zaleplon	C17H15N5O	306.1349
Ziprasidone	C21H21ClN4OS	413.1197
Zolazepam	C15H15FN4O	287.1303

© 2024 Kuai D et al. *JAMA Network Open.*

Zolpidem	C19H21N3O	308.1757
Zonisamide	C8H8N2O3S	213.0328
Zopiclone	C17H17ClN6O3	389.1123

© 2024 Kuai D et al. *JAMA Network Open.*
