# Supplementary material for: Identification and Health Risks of an Emerging Means of Drug Use in Correctional Facilities
Source: JAMA Netw Open. 2024 Dec 23;7(12):e2451951. doi: 10.1001/jamanetworkopen.2024.51951 (PMC11667344; doi:10.1001/jamanetworkopen.2024.51951)
Supplement: Supplement 2. — Data Sharing Statement [file jamanetwopen-e2451951-s002.pdf]

Data Sharing Statement

Kuai. Identification and Health Risks of an Emerging Means of Drug Use in Correctional Facilities. *JAMA Netw Open*. Published December 23, 2024.
doi:10.1001/jamanetworkopen.2024.51951

Data
Data available: Yes
Data types: Deidentified participant data
How to access data: Data will be made available upon reasonable request to the corresponding author (jecarpe@emory.edu).
When available: With publication

Supporting Documents
Document types: None

Additional Information
Who can access the data: Researchers whose proposed use of the data has been approved.
Types of analyses: For further scientific study.
Mechanisms of data availability: After approval of a proposal.
